# DEER and RIDME Measurements of the Nitroxide-Spin Labelled Copper-Bound Amine Oxidase Homodimer from Arthrobacter Globiformis

**DOI:** 10.1007/s00723-021-01321-6

**Published:** 2021-03-29

**Authors:** Hannah Russell, Rachel Stewart, Christopher Prior, Vasily S. Oganesyan, Thembaninkosi G. Gaule, Janet E. Lovett

**Affiliations:** 1 SUPA School of Physics and Astronomy and BSRC, University of St Andrews, St Andrews, KY16 9SS UK; 2 School of Chemistry, University of East Anglia, Norwich, NR4 7TJ UK; 3 School of Molecular and Cellular Biology, Astbury Centre for Structural Molecular Biology, University of Leeds, Leeds, UK

## Abstract

In the study of biological structures, pulse dipolar spectroscopy (PDS) is used to elucidate spin–spin distances at nanometre-scale by measuring dipole–dipole interactions between paramagnetic centres. The PDS methods of Double Electron Electron Resonance (DEER) and Relaxation Induced Dipolar Modulation Enhancement (RIDME) are employed, and their results compared, for the measurement of the dipolar coupling between nitroxide spin labels and copper-II (Cu(II)) paramagnetic centres within the copper amine oxidase from *Arthrobacter globiformis* (AGAO). The distance distribution results obtained indicate that two distinct distances can be measured, with the longer of these at c.a. 5 nm. Conditions for optimising the RIDME experiment such that it may outperform DEER for these long distances are discussed. Modelling methods are used to show that the distances obtained after data analysis are consistent with the structure of AGAO.

**Supplementary Information:**

The online version contains supplementary material available at 10.1007/s00723-021-01321-6.

## Introduction

In the study of biological structures, pulsed dipolar spectroscopy (PDS) can be a valuable tool. The collection of experiments encompassed by PDS measure the magnetic dipole–dipole interaction between centres containing unpaired electrons [1–3], the results of which are commonly used as the basis for extracting nanometre-scale distances, and sometimes orientations, between spin pairs and therefore determining complexes and conformations in biomacromolecules [4]. As only a small population of biological structures contain naturally occurring paramagnetic centres at appropriate sites, most biomolecules typically require the addition of artificial paramagnetic centres through site directed spin labelling (SDSL) to enable PDS studies [5]. Spin labels are molecules that carry a stable paramagnetic centre which can chemically attach to a biomolecule at a site of interest; therefore, allowing PDS measurements to be carried out on these systems [1, 6]. An example spin label, and the one used in this work, is the nitroxide-containing MTSL (S-(2,2,5,5-tetramethyl-2,5-dihydro-1H-pyrrol-3-yl)methylmethanesulfonothioate) which forms a disulfide bond with a free cysteine residue in a protein to yield paramagnetic sidechain R1 [7, 8]. 

Since the R1 linkage is inherently and necessarily flexible to allow efficient chemical conjugation, its use can determine the accuracy of inter-spin distances and orientations extracted from PDS experiments; spin label modelling is therefore a key tool. Sophisticated and computationally demanding molecular dynamics (MD) simulations allow for construction of a protein model from its primary sequence and the addition of the spin label for explicit inclusion in calculations (with the optional inclusion of experimentally derived structural restraints). Such MD simulations enable the time evolution of complex biomolecular systems with introduced spin labels and probes to be monitored and analysed in relation to their EPR spectra [9–11].

There are however various freely available methods for investigating the placement of the labels on a given biomolecular structure through the use of pre-calculated rotamers of spin labels. The programs then calculate/estimate the allowed positions for the paramagnetic centre. Examples of these programs, though not an exhaustive list, are MTSSLWizard, ALLNOX and MMM [12–14]. In this work, we use the open-source Matlab-based multiscale modelling of macromolecular systems (MMM) toolbox which enables visualisation of protein models, spin labels, and their respective conformations [14]. It employs a rotamer library approach, and Lennard–Jones potentials to avoid atom clashes, to enable the prediction of distance distributions between pairs of spins in the model which, in turn, can contribute to informing spin label placement.

Metal ions intrinsic to the biomolecule may have much less uncertainty in position since there are labelling methods which directly bind some metal centres to the amino acids without an intermediate tether. This methodology is at the root of the use of copper(II) (Cu(II)) as a spin label [15–18]. In proteins, a Cu(II) chelate with sub-saturated coordination is bound to a di-histidine motif which may be engineered to the site of interest, just as a cysteine residue can be for MTSL labelling. It has been shown that its use in PDS can yield precise distances which more accurately reflect the backbone conformations of the protein than MTSL can [16]. Cu(II) labelling can also be used in partnership with cysteine labelling (with e.g. MTSL). The use of spectroscopically divergent paramagnetic centres is called orthogonal spin labelling, and it is another weapon in the arsenal of PDS for structural biology since selective information can be garnered from a single sample through exploiting these spectroscopic differences [6].

Double Electron Electron Resonance (DEER, also known as PELDOR) is the most widely used of the PDS techniques, with the four-pulse sequence being most common [19, 20]. The DEER experiment uses two microwave frequencies, one to pump coupled spins, and one to observe the effect via (typically), a refocused Hahn Echo sequence. The dipolar coupling frequency is a modulation on the echo and can be analysed to give distances and distributions [21]. It has also been exploited to give orientation information [22, 23]. Due to the double frequency nature of DEER, it is generally most suited to measuring distances between pairs of identical organic radical labels. This is because other paramagnetic centres tend to have much broader EPR absorption profiles which pose a challenge for the excitation bandwidth of most EPR spectrometers, this includes Cu(II)-Cu(II) measurements [24, 25]. Nitroxide-Cu(II) pairs benefit from the ability to set the pump pulse on the spectroscopically more narrow nitroxide, but there is a caveat that the relative *g*-values separate the spectra such that in practice, frequencies above X-band do not have sufficient resonator bandwidth to allow excitation of both species [26]. Therefore, in measuring interactions between a Cu(II)-nitroxide pair a more recent PDS experiment of Relaxation Induced Dipolar Modulation Enhancement (RIDME, typically the five-pulse sequence) appears to be preferable [27–30].

While DEER uses the microwave-frequency pump pulse to invert coupled spins, RIDME relies on the coupled centre undergoing longitudinal relaxation to modulate the signal of the observed spin centre [1]. The longitudinal relaxation times (*T*_1_) differ substantially between Cu(II) and nitroxides at a given temperature, with Cu(II) being shorter, and thus observing the nitroxide centre’s modulation by the Cu(II) results in a modulated echo signal which can be analysed for distances and distributions. The RIDME experiment has been shown to be significantly more concentration sensitive than DEER for nitroxide-Cu(II) pairs [29, 30]. Both PDS methods measure inter-spin distances in the nanometre range. For DEER, the limit is determined by the phase memory time, related to the spin–spin relaxation time, of the centres [31]. For (spectroscopically) orthogonal labelling where one of the pair is a nitroxide that would typically be the species at the pump frequency, this limit is usually determined by the second paramagnetic centre, e.g. Cu(II). For RIDME, the limit is also determined by the phase memory time of the observed spin, but since this would usually be the nitroxide, it may be less limiting. However, the RIDME signal is also determined by a more complicated and faster decaying so-called “background” function overlaying the modulated echo than in DEER. RIDME time traces typically also have an increased number of artefacts caused by improper phase-cycling of the pulses which can further reduce the quality/reliability of the acquired result. The literature for nitroxide-Cu(II) distance measurements by DEER and RIDME cover the same range of distances with no published data exceeding, to the best of our knowledge, 4 nm [26, 29, 32–35]. 

In this work, we will demonstrate a comparison of DEER and RIDME for a system with two dominant and relatively long nitroxide-Cu(II) distances. To do this, we utilise the copper amine oxidase from *Arthrobacter globiformis* (AGAO). The protein is a homodimer with each monomer containing one Cu(II) in its active site. Previous studies involving this protein have measured the active site coppers using DEER, and also used it as a means of studying orientation selection [31, 36]. The structure of the protein has been determined by X-ray crystallography to 1.8 Å resolution (protein data bank (PDB) code: 1IU7) [37]. The AGAO monomer structure (PDB code: 1W6G), solved to 1.55 Å resolution [38], also shows this Cu(II)-containing active site, as well as a second Cu(II) binding site located on the surface of the protein, approximately 2 nm from the active site Cu(II). Wild-type AGAO has two free cysteines, one of which was mutated to serine (C636), and the other labelled with MTSL (C343) to give the R1 side chain. It is this AGAO-C636S-C343R1 that is used in the paper, and will be called simply AGAO henceforth. The results are promising for extending the limits of DEER and RIDME and comparing their use via typical applications of the four-pulse DEER and five-pulse RIDME on a commercial spectrometer without shaped pulses. However, the distances were not as expected for the holo-enzyme and spin label modelling with MMM and MD was employed to elucidate the reason for this.

## Experimental Methods

### Mutagenesis, Protein Expression, and Purification

The plasmid pETDuet-AGAO (41) was mutated by a site directed mutagenesis procedure using KOD polymerase. The mutagenic primer pairs

$$\begin{gathered} {\text{C636Sfr }}({{5}^\prime} - {\text{CAGTCCGGCTCCCAC}}{\bf{A}}{\text{GCCACGGCAGCGCTTGG}}) \hfill \\ {\text{C636Sr }}({{5}^\prime} - {\text{CCAAGCGCTGCCGTGGC}}{\bf{T}}{\text{GTGGGAGCCGGACTG}}) \hfill \\ \end{gathered}$$

were used to incorporate a single base change (highlighted bold) at Cys (636S) codon from TCG to AGC. DNA sanger sequencing confirmed the generation of pETDuet-C636S (see SI).

The AGAO variant, C636S, was expressed as described in [39]. In brief, BL21 DE3 star cells were transformed with pDUET-C637S. Single colonies were used to inoculate 5 ml of LB media supplemented with 100 μg/ml carbenicillin and grown at 37 °C with shaking at 220 rpm for 16 h. Following this, large scale cultures of LB media supplemented with 100 μg/ml of carbenicillin were inoculated with the starter cultures and grown at 37 °C. Once the culture reached an OD_600_ of 0.6–0.8, IPTG (isopropyl-β-d-thiogalactopyranoside) was added to a final concentration of 1 mM and grown for a further 16 h at 25 °C, 150 rpm. The cell cultures were harvested and lysed. Following lysis, CuSO_4_ was added to the lysate to a final concentration of 1 mM (40). Prior to purification, excess CuSO_4_ was removed by dialysis against phosphate-buffered saline (PBS, 140 mM NaCl, 2.68 mM KCl, 10 mM Na_2_HPO_4_, 2 mM KH_2_PO_4_, pH 7.4). The protein was purified as previously described [40].

Following purification and dialysis against PBS, the protein sample was concentrated to a dimer concentration of 1.38 mM and flash frozen for shipping and storage.

#### Spin-Labelling and Sample Preparation

For the AGAO Q-band measurements, 10 µl of the protein stock was diluted with a mixture containing 49 µl PBS and 1 µl of a 1 mg/40 µl MTSL/DMSO stock. The mixture was left in the refrigerator for 24 h and then washed free of excess spin label and into PBS in deuterium oxide using an Amicon Ultra-0.5 10 kDa cut-off centrifugal filter unit. The final volume was 40 µl. CW EPR was taken (see Fig. S1). 15 µl of this was taken and diluted with a further 15 µl PBS in deuterium oxide.

The AGAO sample for X-band was prepared by taking 15 µl of the protein stock and diluting with 85 µl PBS and 2 µl of a 1 mg/40 µl MTSL/DMSO stock. The reaction was left on the bench for 30 min before having excess label removed and the buffer exchanged for PBS in deuterium oxide and concentrated to 30 µl using the same procedure as above.

Both X- and Q-band samples were finally prepared for EPR by taking 30 µl of the prepared spin-labelled protein and adding 30 µl of glycerol-*d*_8_ before placing in a 3 mm O.D. quartz tube and freezing by placement in liquid nitrogen for the EPR measurements. Assuming no losses during preparation, the Q-band dimer concentration was estimated to be 172 µM and the X-band dimer concentration 0.35 mM.

The AGAO + Cu sample was prepared by adding CuSO_4_ in water to the X-band sample to give an approximate equivalence to monomer, and a 6% overall dilution. The solution was incubated on the bench for 90 min before being syringed back into an EPR tube and frozen for measurement and storage at − 80 °C.

The AGAO + Cu was stored for more than 2 years in the − 80° freezer in the EPR tube before being measured again following thawing and re-freezing. The results in this manuscript are for the protein post storage but they agree with measurement data taken before storage. The (AGAO + Cu)_10xdil_ was made from AGAO + Cu by removing 6 µl and diluting with a 1:1 mixture of PBS in deuterium oxide and glycerol-*d*_8_. The (AGAO + Cu_2_)_10xdil_ was the previous sample with an extra 66 µM copper from a 1 M CuSO_4_ stock in deuterium oxide, diluted to make a 5 mM CuSO_4_ stock (PBS in deuterium oxide) so that 0.8 µl was added to the sample before being returned to the same EPR tube. The (AGAO + Cu_12_)_10xdil_ was the previous sample with an extra 660 µM copper from a 1 M CuSO_4_ stock in deuterium oxide, diluted to make a 50 mM CuSO_4_ stock (PBS in deuterium oxide) so that 0.8 µl was added to the sample before being returned to the same EPR tube.

### EPR Experiments

#### DEER Experimental Methods and Data Analysis

In this work, we utilise three- and four-pulse DEER sequences. The three-pulse experiment followed the sequence (*π*/2)_υ,obs_ − *t* − (*π*)_υ,pump_ − (τ-*t*) − (*π*) _υ,obs_ − τ − echo, while the four-pulse follows the dead-time free sequence (*π*/2)_υ,obs_ − *τ*_1_ − (*π*)_υ,obs_ − (*τ*_1_ + *t*) − (*π*)_υ,pump_ − (*τ*_2_−*t*) − (*π*) _υ,obs_ − * τ*_2_ − echo. In these sequences, the subscripts *υ*,obs and *υ*,pump represent pulses at the observer and pump frequencies, respectively, *t*, the timing of the pump pulse, is incremented while delays represented by some *τ*_n_ are kept constant to achieve a total transverse magnetisation evolution time of 2*τ* for the three-pulse sequence, and 2* τ*_1_ + 2* τ*_2_ for the four-pulse. The echo (or refocussed) intensity is collected over the time window except for the first and final 80 ns to avoid artefacts from the pulses. The first pulse is phase cycled, and for the four-pulse sequence, the *τ*_1_ is stepped to average out any contribution from forbidden hyperfine transitions.

AGAO measurements were recorded at X- and Q-band frequencies on a Bruker ELEXSYS E580. X-band measurements were taken with an MS3 resonator, while the high power (150 W) Q-band measurements utilised the ER 5106QT-2w cylindrical resonator. The DEER experiments were carried out at 50 K and were set up such that the maximum of the nitroxide’s absorption spectrum was the pump frequency position and the observer sequence was offset to higher frequency for Q-band and lower frequency for X-band. The X-band DEER experiment used a four-pulse sequence with *τ*_1_ = 400 ns, *τ*_2_ = 3 μs, Δ*t* = 8 ns and *υ*_obs_–*υ*_pump_ = 65 MHz, where *υ*_obs_ = 9.286 GHz and *υ*_pump_ = 9.221 GHz. The observer pulses had lengths of 32 ns, and the pump pulse was 16 ns. The shot repetition time (SRT) in all cases was determined as being the value at which the echo was at c.a. 70% of its maximum amplitude, which in this case was 4080 μs. The *τ*_1_ was stepped 8 times in increments of 56 ns. The four-pulse sequence was also used in the Q-band experiments with *τ*_1_ = 400 ns, *τ*_2_ = 4 μs, Δt = 8 ns and υ_pump_—υ_obs_ = 80 MHz, with υ_pump_ = 34.010 GHz and υ_obs_ = 33.930 GHz. Here, the SRT was 4080 μs, and the observer pulse lengths were again 32 ns, but the pump pulse was now 14 ns. *τ*_1_ was stepped 5 times in increments of 24 ns.

In the study of the AGAO sample after extra copper was added to the system, which will be referred to as AGAO + Cu, DEER experiments were recorded at 15 K at X-band frequency with both three- and four-pulse sequences. The pump frequency was set to the maximum of the nitroxide’s absorption spectrum and the copper signal was set as the observer frequency. Therefore, the experiment was limited by the phase memory time of the copper and hence the three-pulse DEER experiment was useful to measure the lower dipole–dipole frequencies present. The three-pulse experiment used *τ*_1_ = 120 ns, *τ*_2_ = 2 μs, Δt = 8 ns, SRT = 2040 μs and *υ*_obs_–*υ*_pump_ = 218 MHz, where *υ*_obs_ = 9.446 GHz and *υ*_pump_ = 9.228 GHz. The observer pulses had a length of 32 ns, and a pump pulse of 16 ns. 961 averages were made. The four-pulse experiment used *τ*_2_ = 1.2 μs, which was stepped 8 times in increments of 56 ns and overall 49 scans were averaged together. The total (dead-time free) DEER time trace (called DEERS in this paper) was obtained through utilising DeerStitch [31]. Further details of the process are provided in the SI.

The DEER time trace datasets were analysed using DeerAnalysis2019 [21]. In both the X- and Q-band DEER data, 800 ns was cut from the end to remove distortions present at the end of the spectra [41]. The backgrounds were fitted using a three-dimensional homogeneous distribution model which fits an exponential decay function to the background. For X-band, the background start value was determined by DeerAnalysis and was set to 728 ns, and for Q-band, this value was 544 ns. Likewise, the zero time was also determined by DeerAnalysis and used values of 319 ns and 331 ns for X- and Q-band, respectively. Distance distributions were then extracted from the background corrected spectrum using Tikhonov regularisation. The Tikhonov regularisation parameter was determined by DeerAnalysis according to the L curve criterion and was calculated to be 25.1 for both X- and Q-band.

The DEERS time trace was also analysed in DeerAnalysis2019, but no data were cut prior to analysis. Again, the background was fit using a three-dimensional homogeneous distribution model. DeerAnalysis determined the background start value to be 584 ns with the zero time at 0 ns. The Tikhonov regularisation parameter was 794, as determined by DeerAnalysis according to the L curve criterion.

In all plots showing DEER/DEERS data and associated distance distributions, the intensity depicted is the normalised to the maximum, except the background validation results (Figs. S17 and S18) where DeerAnalysis2019 intensity values are used.

#### RIDME Experimental Methods and Data Analysis

In this work, RIDME experiments were run at Q-band frequency (34 GHz) with a five-pulse sequence of *π*/2 − * τ*_1_ − * π*  − (*τ*_1_ + *t*) − * π*/2 − T_mix_ − * π*/2 − (*τ*_2_-*t*) − * π*  − * τ*_2_ − echo, where the time interval *t* was incremented, while those represented by *τ*_1_, *τ*_2_ and T_mix_ were kept at constant values to obtain an overall magnetisation evolution time of 2* τ*_1_ + 2* τ*_2_ + T_mix_. The refocused virtual echo was collected with a dead-time of 80 ns at the beginning and end of the collection window to avoid artefacts from the pulses.

The AGAO RIDME measurements were performed at a temperature of 25 K (also 50 K, see Figs. S5 and S6) with a *π*/2-pulse length of 16 ns and a *π* -pulse length of 32 ns. The time delays in the sequence had values of *τ*_1_ = 400 ns and *τ*_2_ = 4400 ns, both values were stepped 3 times in increments of 40 ns. The mixing time delay, T_mix_, used for this measurement was 40 μs, and the SRT was 20 ms. AGAO + Cu RIDME measurements were performed at a range of temperatures with the data shown in the main paper at 30 K, with lengths of 32 ns and 16 ns for *π* - and π/2-pulses. The time delay values were *τ*_1_ = 200 ns and *τ*_2_ = 4280 ns, or *τ*_1_ = 200 ns and *τ*_2_ = 6280 ns. The *τ*_1_ and *τ*_2_ were stepped 3 times in increments of 40 ns to average out unwanted effects from nuclear coupling so that each scan consisted of an 8-step phase cycle and the nuclear modulation averaging giving 72 time traces per measurement. A SRT of 8160 μs was used in all the 30 K AGAO + Cu RIDME experiments presented. A variety of T_mix_ values were used and will be provided in the relevant figure captions. Data were collected using a sub-optimal (in terms of amplification) video gain of 18 dB which helped to significantly reduce artefacts at the zero time. For a wider discussion surrounding parameter choices for the RIDME experiments, the reader should refer to section S6 in the accompanying SI.

We used DeerAnalysis2019 to analyse the RIDME data. As before, distance distributions were extracted using Tikhonov regularisation, but it has been suggested that RIDME traces can be fitted using Gaussian functions [42, 43], which is a method that has previously been applied to DEER traces [44, 45]. Unlike DEER, fitting of the RIDME background is not so straight forward. It has been previously noted that due to the stretched exponential decay shape of *T*_1_ relaxation, it is likely that the RIDME background will also have a shape that can be described by a stretched exponential function [46], and in fact, it is found that a stretched exponential function has commonly been used to fit the shape of the RIDME background [47, 48]. It is this background correction process that we adopt, though we show the results from polynomial correction in the SI (Section S7) [49, 50]. In all RIDME data presented herein, the background start was calculated by DeerAnalysis to be the optimal value.

The AGAO RIDME data were cut by 2141 ns prior to background correction to remove severe distortions. The background was fit with a stretched exponential function with a stretch parameter of 5.41 dimensions. DeerAnalysis determined the background start value to be 872 ns, and the zero time to be 341 ns. The Tikhonov regularisation parameter was determined, again by DeerAnalysis, to be 79.4 using the L curve criterion. For the AGAO + Cu RIDME results, no data were cut. The correction to these datasets was carried out by fitting a stretched exponential function to the background (see SI section S7.1 for polynomial background fitting result). For the T_mix_ = 5 μs RIDME data, a stretch parameter, determined by DeerAnalysis, of 2.37 dimensions was used. Also for this dataset, DeerAnalysis determined the optimal background start value to be 2216 ns, and the zero time was 136 ns. The Tikhonov regularisation parameter was determined by DeerAnalysis according to the L curve criterion and had a value of 398. For the T_mix_ = 10 μs RIDME data, a stretch parameter of 3.68, background start of 784 ns, zero time of 138 ns, and Tikhonov regularisation parameter of 631, were all determined by DeerAnalysis to be optimal.

In all plots showing RIDME data and associated distance distributions, the intensity depicted is the normalised to the maximum, except the background validation results (Figs. S17 and S18) where DeerAnalysis2019 intensity values are used.

#### Other EPR Experiments

Experimental details and results for room temperature CW EPR, inversion recovery *T*_1_, phase memory and echo-detected field swept spectra are given in the SI. All plotted data are normalised to the maximum intensity.

### Modelling

#### MD Simulations

All MD simulations were performed using the AMBER16 software package [51]. The starting configuration was generated by replacing the relevant cysteines with the spin label using the MMM program [14] in the crystal structure of 1IU7 from the protein databank [37]. Further, three amino acids were removed from the end of both the A and B-chains of the protein structure. These amino acids are proline, alanine, and asparagine. The reason for their removal is the obstruction they cause to the attached MTSL which was shown by preliminary MMM results (not shown) and also the true protein sequence was longer, and so this was not the true C-terminal sequence.

The protein was modelled using the FF14SB force field [52] and extended to the TPQ residue using additional parameters from the General Amber Force field (GAFF) [53]. MTSL spin label was modelled using force field and topology parameters developed previously [9]. Water was treated using SPC/E model [54] with the associated ion parameters [55].

An NPT ensemble was maintained at a pressure of 1 atm using the Berendsen algorithm [56] with a coupling constant of 5 ps at 273 K. The SHAKE algorithm [57] was used to maintain the hydrogen bond lengths. The centre of mass motion was removed every 20 ps and a time step of 2 fs was used. Long range electrostatic interactions were accounted for using the Particle Mesh Ewald [58] method with a cut-off radius of 10 A. The system was equilibrated for 25 ns prior to a production run of 100 ns. During the production run, the structural RMSD was low, indicating the structure was stable and underwent no major conformational changes over the course of the simulation.

#### Using the MMM Software for Spin-Labelling

Prior to analysis, the MD-derived AGAO model was opened in PyMOL [59] where Cu(II) ions were positioned on the structure. Their placement was guided by the crystal structures of PDB: 1IU7 and 1W6G, which provided the active site and surface site Cu(II) positioning, respectively. The MD model also contained CYL sites, that is spin labels bound to cysteine sites, but this site-type cannot be read by MMM. For this reason, the CYL sites were removed in PyMOL and cysteine (CYS) sites were put in their place to allow MMM labelling to be used. The coordinates of the MD-derived AGAO model were then imported to MMM (2018.2 version) which was run with MATLAB R2017b. The A and B chains of the homodimer are numbered as in PDB: 1IU7 and start at residue number 1, which is shifted relative to the true protein N-terminus. The chains are fused into a single chain in the MD model, and so the numbering of the amino acids in chain B is a continuation of the chain A numbering. The R1 rotamer library [60], at 298 K, was used to label the appropriate sites (Chain A: 307Cys and Chain B: 924Cys). While in reality, the spin-labelled protein would exist at lower temperatures, 298 K has been shown to be the recommended temperature for calculation as it was reported that comparison of MMM results measured at this temperature to those obtained experimentally showed 298 K to provide computations that better agreed with the experiment than if the calculations were run at cryogenic temperature [61].

Through these calculations, MMM determined that R1 bound to site 307Cys consists of 70 populated rotamers, which have a root mean square deviation (rmsd) of 0.50 nm and a partition function, *Z* = 1.42443. Meanwhile, MMM finds R1 on site 924Cys to have 53 populated rotamers that make up the 99.5% of the overall weighting, with a rmsd of 0.47 nm and *Z* = 0.65494. MMM was then used to simulate a DEER experiment to extract the expected distance distribution between the attached labels and the Cu(II) centres.

## Results and Discussion

### General Comment

**Fig. 1:**
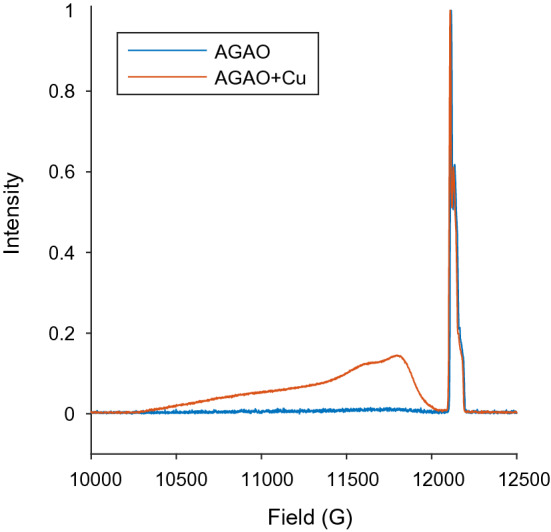
Echo-detected field swept spectra of the AGAO protein samples before and after extra Cu(II) was added to the system. The result shows the original AGAO sample (before Cu(II) was added) to be an essentially apo-protein, while the AGAO + Cu field sweep clearly shows Cu(II)

The protein was successfully expressed, purified and spin labelled. Room temperature CW for the nitroxide is shown in Fig. S1. To avoid odd results which were highly asymmetric across the two monomers of the homodimer and arose from restricted spin label rotamers, the final PAN sequence (see also the SI section S1) from the 1IU7 crystal structure was removed prior to computer simulations of the R1 position. MD was used to relax the protein in silico and to add the R1 side chain. MMM was also used on the relaxed AGAO homodimer structure. The AGAO echo-detected field swept data revealed that despite incubation in Cu(II) prior to protein purification, there was not much copper present, see Fig. 1, as a result, copper was added directly to the EPR sample to give AGAO + Cu sample. It is likely that the process of spin labelling and buffer exchange led to a leaching of the Cu(II) from the protein. The parameters used to record the echo-detected field swept data can be found in the SI. One effect of adding the copper was to limit the signal of the nitroxide through a reduction of its phase memory time and this made nitroxide–nitroxide PDS very difficult. Hence, nitroxide-nitroxide PDS results are presented for the AGAO sample whereas nitroxide-Cu(II) PDS results are from the AGAO + Cu.

### Nitroxide–Nitroxide Distance Measurements

**Fig. 2:**
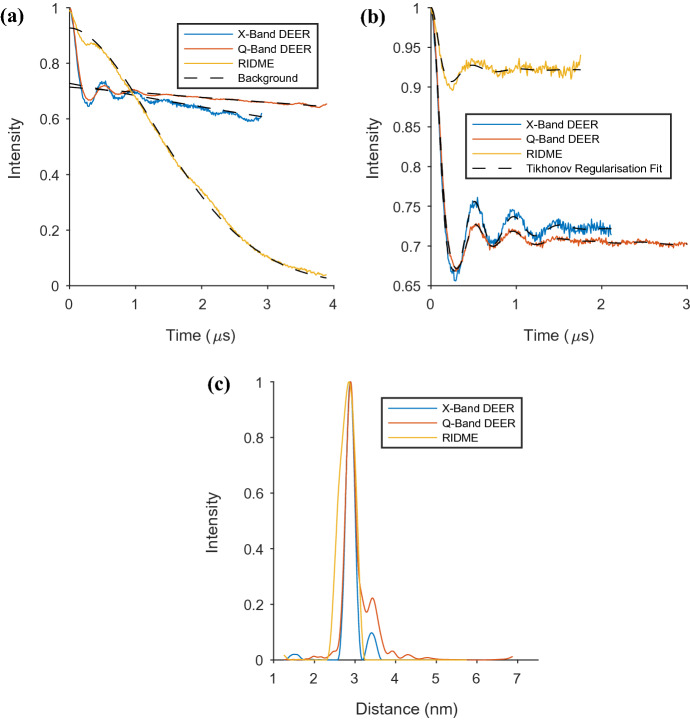
The results of DEER and RIDME measurements on the AGAO sample prior to extra Cu(II) being added to the system are presented. Both the DEER and RIDME experiments were run at a temperature of 25 K. The RIDME experiment used Q-band frequency, while the DEER experiments were run at both X and Q-band. The RIDME experiment used a T_mix_ of 40 μs; **a** raw data for DEER and RIDME measurements. The background of each spectrum is also presented as a dashed line on these plots; **b** background corrected data again for DEER and RIDME. Here, the Tikhonov regularisation fit is also presented as a dashed line on each spectrum; **c** distance distributions found from DEER and RIDME measurements after background correction and using Tikhonov regularisation methods

DEER and RIDME measurements were conducted on the sample, and the results are shown in Fig. 2. The DEER experiments were run at two frequencies; X-band and Q-band. The modulation depths are similar for X- and Q-band which is likely a coincidental effect of differing concentrations, EPR frequency and pump pulse length. They are however consistent with approximately full double spin labelling of the AGAO. The extracted distance distribution from each measurement showed the results to be in very good agreement, though with some difference at longer distance which remains despite validation using DeerAnalysis2019 (Fig. S17). The samples were not identical in concentration and were not spin labelled at the same time, so the origin of this minor difference could be from the sample, or in the difference in the EPR frequency between the two results. However, this is a minor component and we do not consider its presence or absence to alter the conclusions of this work.

The acquired RIDME AGAO distance distributions are plotted alongside those from DEER in Fig. 2c. Comparing the DEER and RIDME results, we see that they are largely in agreement, with only minor differences. Here, we present RIDME data measured at a temperature of 25 K (see Figs. S5 and S6 for the result at 50 K). The modulation depth from RIDME is smaller than measured in DEER for this nitroxide–nitroxide system (Figs. 2a, b). DEER’s use of observer and pump pulses of two different frequencies provides the technique’s advantage here as a greater number of nitroxide spins are pumped than those inverted naturally during the mixing time used in the RIDME experiment.

### Nitroxide-Cu(II) Distance Measurements

**Fig. 3:**
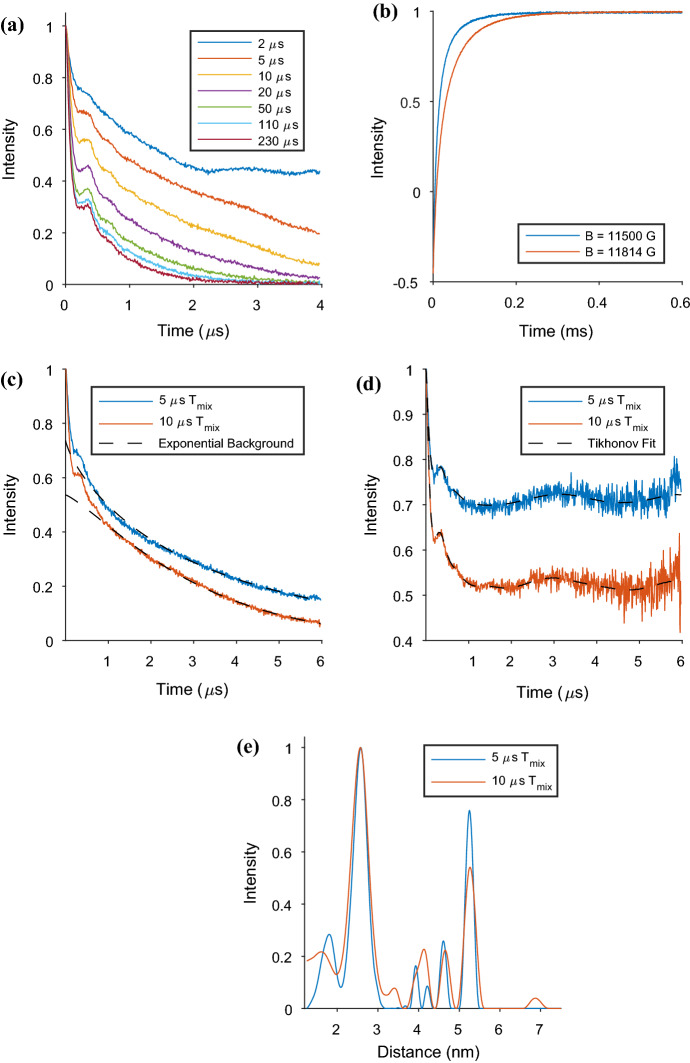
RIDME measurements, all measured at 30 K on the AGAO + Cu sample, with different mixing time values and collection window time lengths; **a** raw data collected from RIDME measurements on the AGAO + Cu sample, all measured at 30 K with *τ*_2_ = 4280 ns, with mixing time values ranging from 2 μs to 230 μs; **b** inversion recovery curves measured on the Cu(II) spins at two field positions (11,500 G and 11,814 G), the corresponding echo decays are in Fig. S4; **c** raw data of RIDME experiments measured at two mixing times, 5 μs and 10 μs, with *τ*_2_ = 6280 ns. The accompanying background, fit with a stretched exponential function, are also presented; **d** is the background corrected RIDME traces of the data shown in **c** with Tikhonov regularisation fit, and **e** distance distributions extracted from both measurements

RIDME measurements were conducted using a series of different mixing time values ranging from 2 μs to 230 μs at 30 K. The raw data from a selection of these measurements are presented in Fig. 3a.

From these data, we observe that the modulation depth increases, and therefore improves, with lengthening mixing time. This is expected from the *T*_1_ relaxation time of the copper (see Fig. 3b, and SI for experimental parameters). This is because an optimal modulation depth is obtained with mixing times close to the time for full inversion of the coupled Cu(II) centre. The theoretical limit of a nitroxide-Cu(II) RIDME experiment modulation depth is 50% [27]. Here, however, we have a system that has two nitroxides which could be interacting with up to four Cu(II) centres each, and in Fig. 3a, we observe a modulation depth greater than 50% for the longer mixing times. As the mixing time values become greater, we observe that the rate at which the RIDME background decays to zero increases. The reason for RIDME’s acute background decay is due to hyperfine spin diffusion and the presence of protons in the protein and/or matrix which increase the rate of the relaxation process [62]. Replacing protons by deuterons decreases the severe background decay [46]. The background decay limits the available data collection time window and hampers the fitting of a background function by DeerAnalysis. Here, we show that this background is highly dependent on the mixing time. In the SI, we show that it is not particularly dependent upon temperature (Fig. S8), though the signal-to-noise depends upon the phase memory time of the nitroxide and consequently decreases with increasing temperature.

The background causes issues in using DeerAnalysis for background correction but, more critically for accurate distance distribution profile determination, causes the dipolar modulations to be damped. This is exemplified in Fig. 3c–e that show the raw RIDME time traces, background corrected traces, and extracted distance distributions, respectively, from two of the datasets with shorter mixing times (5 μs and 10 μs experiments, which were measured to better signal-to-noise, and with a *τ*_2_ 2 μs longer, than the data in Fig. 3a). We again observe the increase in modulation depth with mixing time that we saw in Fig. 3a. However, the analysis of the data clearly shows that there are at least two measurable distances in this system (as expected), with the two most prominent being a shorter one with a modal distance of 2.6 nm and a longer one at about 5.1 nm. The 5 and 10 μs RIDME experiments presented in Fig. 3c–e are compared to the distance distribution results from fitting the background with a second-order polynomial function in the SI (Figs. S13 and S14). The results from free fitting two or three Gaussians to the time traces are shown in Figs. S15 and S16, with a discussion of results in section S7.2. Inspection of the combination of results is consistent with the Tikhonov derived distribution presented in Fig. 3c. The distance distributions in Fig. 3e clearly show that the increase in modulation depth is to the detriment of accurate measurement of particularly the longer distance.

By comparison of the 2 μs mixing time raw data to that obtained from other mixing times, obvious artefacts and distortions are observed that make no appearance in any of the other data sets. Therefore, the decision was made to focus attention on a mixing time of 5 μs.

In analysing the data acquired by the DEER experiment on the same sample, the DeerStitch method was required due to the phase memory time of the Cu(II) being too short to measure the long distances we aim to observe [31]. It should be noted that other DEER sequences, including five- and seven-pulse, are able to produce the same result of a longer phase memory time, but that these methods work significantly better with shaped pulses [63, 64]. Therefore, due to the simplicity of DeerStitch method, with the available rectangular pulses, it was employed here.

**Fig. 4:**
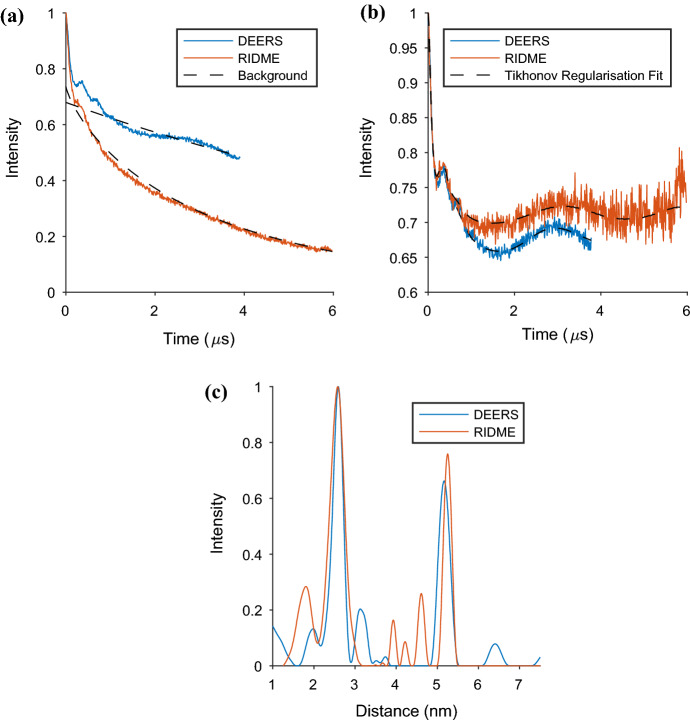
The results of the ‘best’ DEER and RIDME measurements, selected based on the criteria discussed in the main text, on the AGAO + Cu are presented. Both the three and four-pulse DEER experiments, stitched together into a DEERS spectrum, were run at a temperature of 15 K at X-Band frequency, while the RIDME experiment was run at 30 K with T_mix_ = 5 μs; **a** raw data for DEER and RIDME measurements. The background of each spectrum is also presented as a dashed line on these plots; **b** background corrected data again for DEER and RIDME. Here, the Tikhonov regularisation fit is also presented as a dashed line on each spectrum; **c** distance distributions found from DEER and RIDME measurements after background correction and using Tikhonov regularisation methods

Looking at the raw and background corrected DEERS traces (Fig. 4a, b, also Fig. S7), we observe it to be largely free from distortions and artefacts, thereby increasing confidence in obtaining accurate and valid distance values. The distance distribution obtained from DeerAnalysis is shown in Fig. 4c and contains two major distances that are robust to validation using the background start value (see Fig. S18). Figure 4c compares the DEERS to the RIDME result for T_mix_ = 5 μs. The two most prominent distances overlap well in both intensity and width. Note that the background validation of the RIDME experiment (Fig. S18) does not give any certainty to the longer distance peaks. We know through a comparison of the DEERS and RIDME distance distributions, as well as visual inspection of the raw data, that there are two prominent distances measurable in this system. Therefore, this shows that validation through background start value checking in DeerAnalysis for data with an extreme background function may lead to additional uncertainty on longer distance peaks.

RIDME has been shown to have a significant concentration sensitivity, [30] and we were able to repeat elements of a data collection on a sample that was diluted by 10 times with ease. We did not test this sample with the DEER or DEERS experiments but it would be unlikely that it could be measured well, especially since we would need to measure at X-band frequencies. These data are presented in the SI (Fig. S10) along with some tests of adding more copper (Fig. S11) which resulted in some further increase in modulation depth, but a decrease in the phase memory time of the nitroxide, and consequently larger noise levels. We did not analyse the data further.

### Comparison to Protein Model

**Fig. 5:**
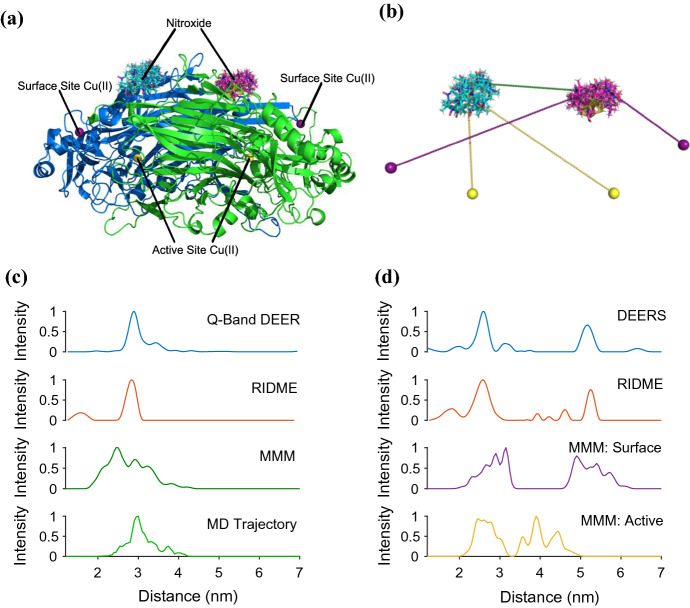
Distance result analysis in terms of AGAO protein structure; **a** shows the MD-derived AGAO model with MMM-calculated R1 rotamers. The active and surface site Cu(II) ions are labelled. In **b**, a schematic of the distance measurements between R1 and Cu(II) centres is presented. The following two panels show the distance distributions extracted using Tikhonov regularisation methods from background corrected RIDME and Q-band DEER measurements compared to MMM calculations for **c** AGAO sample prior to extra Cu being added to the system and **d** AGAO + Cu sample

All distance distributions in this work have been carried out on the equilibrated from MD solution structure of spin labelled AGAO using MMM with rotamer library approach developed for R1 at ambient temperature (Fig. 5a, b) [14]. Calculated nitroxide–nitroxide distances are presented in Fig. 5c as a green line and show good agreement with the nitroxide–nitroxide distances calculated from the 100 ns MD trajectory which has the mean nitroxide–nitroxide distance of 3.10 nm, with a standard deviation of 0.37 nm (see also Fig. S19).

The MMM result for the nitroxide–nitroxide distance has a mean distance of 2.75 nm with a standard deviation of 0.49 nm. Figure 5c compares the MMM prediction to the distance distributions extracted from the DEER and RIDME data on AGAO. Experimentally, the Q-band DEER peak, shown to span the distance between 2.25 nm and 3.75 nm, gave a mean distance of 3.00 nm with an overall standard deviation of 0.26 nm, with a most probable distance of 2.90 nm. The RIDME result agreed well, showing an overall mean distribution distance of 2.89 nm with a standard deviation of 0.96 nm. The peak with the most probable distance of 2.84 nm had a mean of 2.82 nm and a standard deviation of 0.11 nm. These results therefore provide us with a DEER modal value of 2.90 nm and a RIDME mode of 2.84 nm, which are in very good agreement. These values are also within the standard deviation of the mean MMM result.

Figure 5d presents the distance distributions from the DEERS and RIDME experimentation methods used in the measurement of the AGAO + Cu sample. The MMM distribution prediction plotted alongside was calculated by considering only the distances between the spin label and Cu(II) centres; the nitroxide–nitroxide and Cu(II)-Cu(II) interactions were subtracted from the presented results. In Fig. 5d, the protein model distance distribution shows the distances between R1 rotamers and Cu(II) ions bound to AGAO active sites, and the distances between the Cu(II) ions bound to AGAO surface sites and the R1 rotamers, while Fig. 5b presents a schematic of these distance measurements. Both models lead to a collection of peaks that form two distinct groups. The distance from R1 on the A or B-chain of the model to the Cu(II) bound to the active site of the same chain has a mean distance of 2.74 nm with a standard deviation of 0.26 nm, while that between R1 on the A or B-chain and the Cu(II) bound to the surface site of the same chain has a mean distance of 2.85 nm with a standard deviation of 0.32 nm. The longer distances show R1 on one chain and Cu(II) bound to either the active or surface site on the other chain. For R1 to active-site Cu(II) on the other chain, the mean distance is 4.21 nm which has a standard deviation of 0.32 nm, and for R1 to surface-site Cu(II) on the other chain, the mean is 5.35 nm, with a standard deviation of 0.32 nm.

The DEERS and RIDME experiments for AGAO + Cu provided distance distributions with two predominant peaks. This implies that the homodimeric system usually has up to one copper bound in each monomer. In the DEERS results, the first distance peak has a most probable separation of 2.59 nm, where the prominent peak between 2.15 nm and 2.90 nm has a mean of 2.56 nm and standard deviation of 0.14 nm. The second, longer distance peak has a mean of 5.17 nm, where the most probable distance is 5.17 nm with a standard deviation of 0.12 nm. In the RIDME result, we first observe an intense distribution peak that provides a most probable distance of 2.57 nm, where the prominent peak between 2.10 nm and 3.10 nm has a mean distance of 2.57 nm and standard deviation of 0.18 nm, which is almost identical to that obtained via the DEERS experiment. The second prominent peak, with a most probable distance of 5.31 nm, a mean distance of 5.25 nm, and a standard deviation of 0.09 nm, is in good agreement with the DEERS measurement for this separation.

Comparing to the MD protein model MMM-derived distributions, the shortest distance main peak corresponds to the distance between a nitroxide spin and a Cu(II) ion, but whether that Cu(II) is bound to an active or a surface site of the protein located on the same chain as the MTS label is positioned is initially unclear. The longer distance of the second PDS-derived peak can clearly be attributed to that between a nitroxide spin label on one chain and a Cu(II) centre bound to a site on the other protein chain.

Our results support a conclusion that there is no, or negligible, Cu(II) binding occurring at the active sites, and instead Cu(II) is binding to the surface site positions. This is consistent with the PDS distance distribution peaks for the two prominent short and long distances. It is presumably a consequence of adding stoichiometric copper to the spin labelled AGAO, rather than soaking in excess Cu(II) ions and then dialysing away the excess, as in the original preparation procedure. However, on closer inspection, also present in the RIDME distribution are two smaller peaks situated between ~ 4 and 5 nm. We do not wish to overinterpret the data, but note that it cannot be ruled out that during freeze-thawing events that occurred between the DEERS and RIDME experiments the Cu(II) ions may have re-equilibrated and resulted in some partial binding to the active sites of the AGAO protein. Additionally, given the similarity of the shorter main distance to the nitroxide–nitroxide distance (see Fig. 2), and that this signal may not be negligible in the RIDME even at low temperatures (see Fig. S8), we must conclude that the shorter distance may be distorted by an underlying unwanted (nitroxide–nitroxide) signal. Combining the possible effects of secondary copper binding site and artefactual nitroxide–nitroxide measurement, the expected ratio of 1:1 for the area of the shorter and longer peaks in accordance with the expected homodimeric AGAO system, may be even better recovered than can be reported here. This is despite the limiting factors of the background function on the available collection window for the data, and on the accurate recovery of the low frequency dipole–dipole modulations from the RIDME time trace. The second DEERS distribution peak is also not as intense as the first and we attribute this to the time trace length, which is determined by the phase memory time of the copper, being limiting for accurate long distance distribution measurement.

## Concluding Remarks

This work has demonstrated the applicability of the PDS methods DEER and RIDME in the measurement between nitroxide R1 spin centres and bound paramagnetic Cu(II) ions in the AGAO homodimeric protein. Both PDS methods achieved results which were in excellent agreement with each other, and also with an AGAO model produced by MD simulation and the rotamer library approach. Through optimisation of the measurement temperature, mixing time value and careful data processing for the RIDME experiment to avoid strong background effects which tend to dampen the lower dipole–dipole frequencies, the accuracy of the distance distributions from the PDS methods is shown to be comparable. However, RIDME experiments have a distinct advantage over DEER methods in these nitroxide-Cu(II) systems: they are taken at Q rather than X-band which offers greater concentration sensitivity/shorter measurement times; use the longer phase memory echo from the nitroxide rather than the copper which may give a longer measurement window and therefore better measurement of low dipolar frequencies/long distances; and are not sensitive to excess unbound copper [30].

There were two major groups of distances measured by DEERS and RIDME experiments of AGAO + Cu and these were attributed to the separation between an R1 spin centre on one chain of the AGAO protein, and a Cu(II) bound to a surface site on either the same chain (shorter distance) or on the partner protein (longer distance). The longer distance was the important one for determining the surface, rather than active site, binding and the distance was measured by DEERS to have a most probable value of 5.17 nm, and RIDME was in good agreement showing a most probable separation of 5.31 nm, where the MMM-calculated distance was 5.35 nm.

To the best of our knowledge, no published data exceeds a nitroxide-Cu(II) RIDME or DEER measurement greater than 4 nm [26, 29, 32–35], thereby making these measurements the longest of their type as well as demonstrating that multiple distances can be measured.

## Further Information

Supporting information and research data underpinning the figures and conclusions in this publication can be accessed at https://doi.org/10.17630/da9186bb-8b3c-4c6d-9483-88a2bb94731f

## Supplementary Information

Below is the link to the electronic supplementary material.

Supplementary file1 (PDF 1199 KB)
