# Supplementary material for: DEER and RIDME Measurements of the Nitroxide-Spin Labelled Copper-Bound Amine Oxidase Homodimer from Arthrobacter Globiformis
Source: Appl Magn Reson. 2021 Mar 29;52(8):995–1015. doi: 10.1007/s00723-021-01321-6 (PMC8550341; doi:10.1007/s00723-021-01321-6)
Supplement: Supplementary file 1 — Supplementary file1 (PDF 1199 KB) [file 723_2021_1321_MOESM1_ESM.pdf]

# Supplementary Information

# DEER and RIDME Measurements of the Nitroxide-Spin Labelled Copper-Bound Amine Oxidase Homodimer from Arthrobacter Globiformis.

**Hannah Russell[1], Rachel Stewart[1], Christopher Prior[2], Vasily S. Oganesyan[2], Thembaninkosi G. Gaule[3], Janet E. Lovett[1]**

**Addresses**

**[1] SUPA School of Physics and Astronomy and BSRC, University of St Andrews, St Andrews, KY16 9SS, UK;**

**[2] School of Chemistry, University of East Anglia, Norwich, NR4 7TJ, UK.**

**[3] School of Molecular and Cellular Biology, Astbury Centre for Structural Molecular Biology, University of Leeds, Leeds, UK.**

**Table of Contents**

**S1. AGAO-C636S amino acid sequence (pETDUET-C636)……………………………..…2**

**S2. Continuous Wave EPR……………………………………………………………..….2**

**S3. Relaxation and Echo-Detected Field Sweep Measurements ………………………..….3**

**S4. AGAO RIDME Measurements…………………………………………………...…..5**

**S5. AGAO+Cu DEERS Measurements …………………………………….……….6**

**S6. AGAO+Cu RIDME Measurements…………………………………………….…….7**

**S6.1 Effect of Temperature on Measurements……………………………….……..7**

**S6.2 Sample Dilution………………………………………………………………9**

**S6.3 Effect of Increasing Cu(II) Concentration………………………………….…...11**

**S7. Alternative RIDME Fitting Methods………………………….……………………..…13**

**S7.1 Stretched Exponential vs Polynomial Background Fitting…………………..…13**

**S7.2 Tikhonov Regularisation vs Gaussian Distribution Analysis…………..……17**

**S8. Background Validation Data……………………………………………………….…..19**

**S9. MD Simulation AGAO Modelling……………………………………………….……..20**

## S1. AGAO-C636S amino acid sequence (pETDUET-C636)

M**G**TPSTIQTA SPFRLASAGE ISEVQGILRT AGLLGPEKRI AYLGVLDPAR GAGSEAEDRR<br>
FRVFIHDVSG ARPQEVTVSV TNGTVISAVE LDTAATGELP VLEEEFEVVE QLLATDERWL<br>
KALAARNLDV SKVRVAPLSA GVFEYAEERG RRILRGLAFV QDFPEDSAWA HPVDGLVAYV<br>
DVVSKEVTRV IDTGVFPVPA EHGNYTDPEL TGPLRTTQKP ISITQPEGPS FTVTGGNHIE<br>
WEKWSLDVGF DVREGVVLHN IAFRDGDRLR PIINRASIAE MVVPYGDPSP IRSWQNYFDT<br>
GEYLVGQYAN SLELGCDCLG DITYLSPVIS DAFGNPREIR NGI**C**MHEEDW GILAKHSDLW<br>
SGINYTRRNR RMVISFFTTI GNYDYGFYWY LYLDGTIEFE AKATGVVFTS AFPEGGSDNI<br>
SQLAPGLGAP FHQHIFSARL DMAIDGFTNR VEEEDVVRQT MGPGNERGNA FSRKRTVLTR<br>
ESEAVREADA RTGRTWIISN PESKNRLNEP VGYKLHAHNQ PTLLADPGSS IARRAAFATK<br>
DLWVTRYADD ERYPTGDFVN QHSGGAGLPS YIAQDRDIDG QDIVVWHTFG LTHFPRVEDW<br>
PIMPVDTVGF KLRPEGFFDR SPVLDVPANP SQSGSH**S**HGS AWSHPQFEK

The sequence of the AGAO monomer is P46881 in Uniprot. The sequence used here is shifted by one residue due to the glycine inserted at residue number 2 (bold and highlighted in yellow). The free cysteine residue which was labelled with MTSL is at position 343 in the wild type (344 here, shown in bold and highlighted with green) and the serine (shown in bold, highlighted red) is a cysteine in the wild type at position 636. The wild-type numbering is used in the manuscript. The PAN sequence which represents the end of the 1iu7.pdb structure and was required to be removed for the MD and MMM rotamer library approach is underlined.

## S2. Continuous Wave EPR

Room temperature continuous wave (CW) EPR measurement (Fig. S1) was carried out using an Active Spectrum Micro ESR X-band (9.8 GHz) spectrometer. The sample was post-spin labelling and before glycerol was added and was therefore approximated as 1.38 mM. It filled the cavity and was in a Blaubrand micropipette capillary tube. The microwave power was 10 mW and the measurement was averaged over 10 scans. The result shows the MTS-labelling to be successful on the AGAO sample and implies that excess free label was sufficiently washed from the sample.

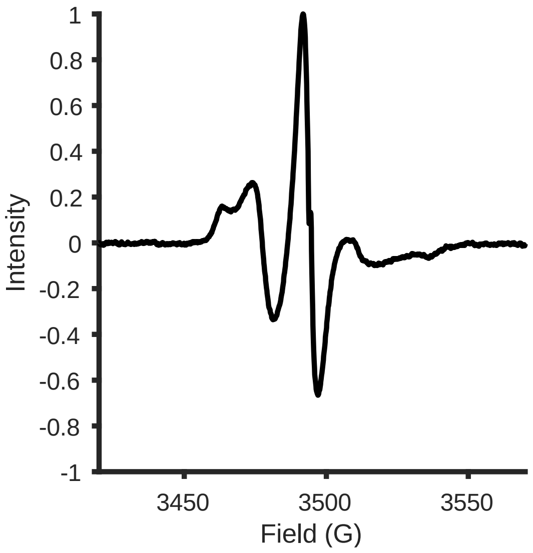


**Fig. S1** Room-temperature continuous wave (CW) EPR spectrum

## S3. Relaxation and Echo-Detected Field Sweep Measurements

The echo-detected field sweep (ED-FS) data was recorded at Q-band frequency, and at temperatures of 25 K and 30 K for the AGAO and AGAO+Cu samples, respectively, using the two-pulse sequence $\pi/2 - \tau - \pi - \tau -$ echo. For the AGAO sample ED-FS, a shot repetition time (SRT) of 100 ms was used, and the $\pi/2$- and $\pi$-pulses were of lengths 16 and 32 ns, respectively. A time delay, $\tau$, of 200 ns was set, and the centre field was 11000 G with a sweep width of 3000 G. For the AGAO+Cu sample, a SRT of 75 ms, pulse lengths of 16 and 32 ns for $\pi/2$- and $\pi$-pulses, respectively, and $\tau$ = 200 ns were all used. The centre field was set to 11200 G with a sweep width of 3000 G. Results are shown in Fig. 1 and Fig. S2.

The inversion-recovery for AGAO+Cu, which indicates $T_1$, was measured at field positions indicated by coloured dots on the ED-FS in Fig. S2. The pulse sequence $\pi - T - \pi/2 - \tau - \pi - \tau -$ echo was used with the pulse lengths of 32 and 16 ns for the $\pi$- and $\pi/2$-pulses, respectively, and time delays $\tau$ = 200 ns and T = 400 ns, which was stepped in increments of 300 ns for B = 11500 G and 11814 G, and 10 μs for B = 12109 G (the nitroxide). For measurement of the rate of $T_2$ relaxation in AGAO+Cu, the two-pulse sequence $\pi/2 - \tau - \pi - \tau -$ echo was used, where the $\pi/2$- and $\pi$-pulse lengths were 16 and 32 ns, respectively, and the time delay was $\tau$ = 200 ns stepped in increments of 10 ns in both the B = 11814 G and 12109 G measurements. Figs. S3 and S4 show the $T_1$ and $T_2$ measurement curves at 30 K.

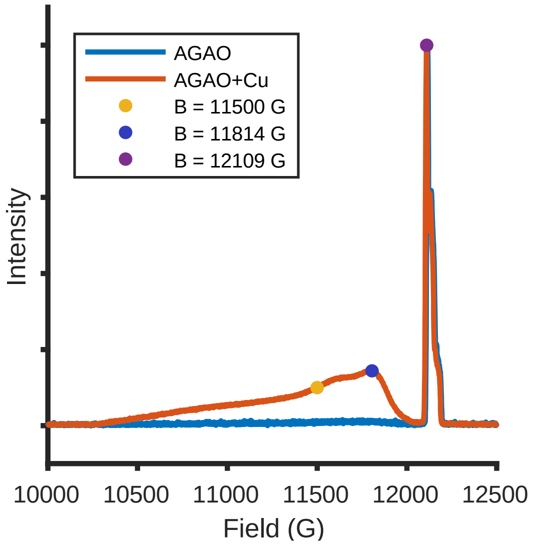


**Fig. S2** ED-FS of the AGAO and AGAO+Cu samples. The coloured points depict field values shown in the legend at which the spin relaxation times were recorded

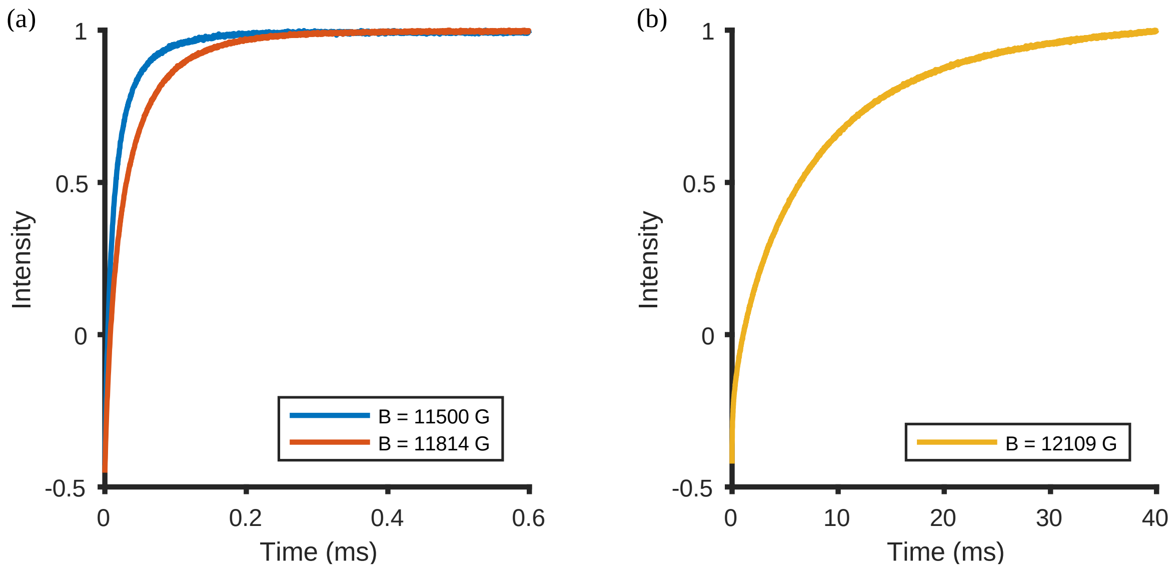


**Fig. S3** Inversion-recovery results measured using the three-pulse inversion recovery experiment for AGAO+Cu at 30 K. The field positions 11500 G, 11814 G, and 12109 G are indicated in the ED-FS in Fig. S2

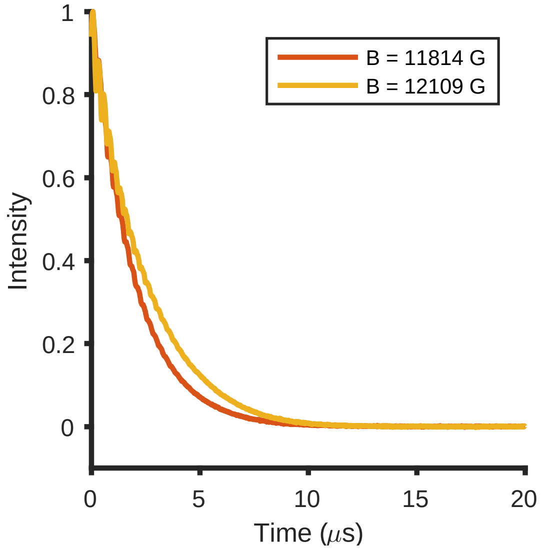


**Fig. S4** Echo-decay measured using a two-pulse experiment for AGAO+Cu at 30 K. The field positions are indicated on the ED-FS in Fig. S2

## S4. AGAO RIDME Measurements

Fig. S5a shows the RIDME data for AGAO measured at two temperatures, 25 K and 50 K, at Q-band frequency. The 50 K RIDME used π/2- and π-pulse lengths of 16 and 32 ns, respectively. $T_{mix}$ was 40 μs, and time delays of $\tau_1$ = 400 ns and $\tau_2$ = 4400 ns, which were stepped 3 times in increments of 40 ns, were used. A SRT of 2550 μs was also set. The 25 K experiment was run with the same parameters, but here a SRT of 20 ms was used. The 25 K data is shown to have been subject to large distortions at about 2 μs, which we attribute to artefacts from insufficient phase correction in the RIDME pulse sequence. However, it can be seen that despite the very large difference in spin-lattice ($T_1$) recovery for the nitroxide at these two temperatures (Fig. S5b), the modulation of the RIDME signal has about the same intensity. The 25 K $T_1$ measurements used the three-pulse inversion recovery experiment, which had π- and π/2-pulse lengths of 32 and 16 ns, respectively, and used time delays τ = 200 ns and T = 400 ns, which was stepped in increments of 90 μs. The measurements used a SRT of 200 ms, and were recorded at the peak nitroxide field of B = 12117 G. The 50 K measurement parameters were identical except for decreased values for the increment size and SRT, which were 40 μs and 60 ms, respectively.

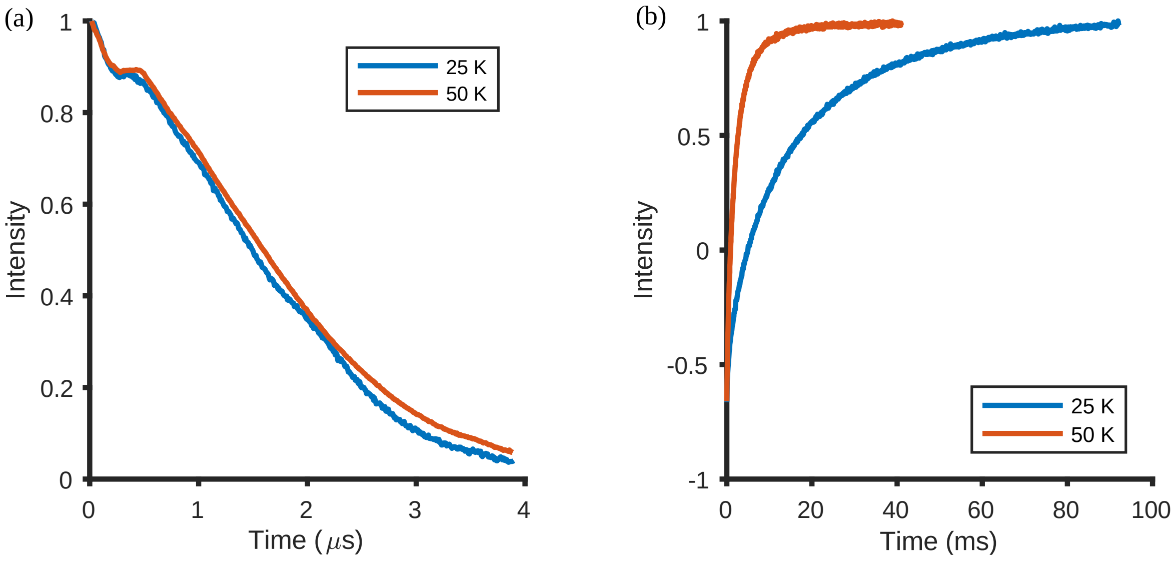


**Fig. S5** AGAO nitroxide-nitroxide RIDME results at 25 K and 50 K: (a) raw data; (b) the $T_1$ relaxation rates of the nitroxide spins, recorded using the inversion recovery experiment, at the two temperatures at the maximum of the nitroxide ED-FS signal

Although the 25 K data suffers from large distortions at longer times, the nitroxide-nitroxide distance separation we expect to obtain from these measurements exist at much shorter times which are not subject to such artefacts. Fig. S6 presents both the 25 K and 50 K data cut by 2141 ns and 2130 ns, respectively, to 1750 ns. For the 25 K data, the background correction was achieved by fitting a stretched exponential with a DeerAnalysis-determined stretch parameter of 5.41 dimensions. Here, the zero time was 341 ns, and the background start value was determined by DeerAnalysis to be 872 ns. The distance distributions were extracted using a Tikhonov regularisation parameter of 79.4, as determined by DeerAnalysis using the L-curve criterion. For the 50 K data, a zero time of 341 ns and background start value of 312 ns were used to fit a stretch exponential to the RIDME data with a stretch parameter of 5.23 dimensions. The distance distribution was obtained using a Tikhonov regularisation parameter of 25.1 using the L-curve criterion. Again, all values were determined by DeerAnalysis.

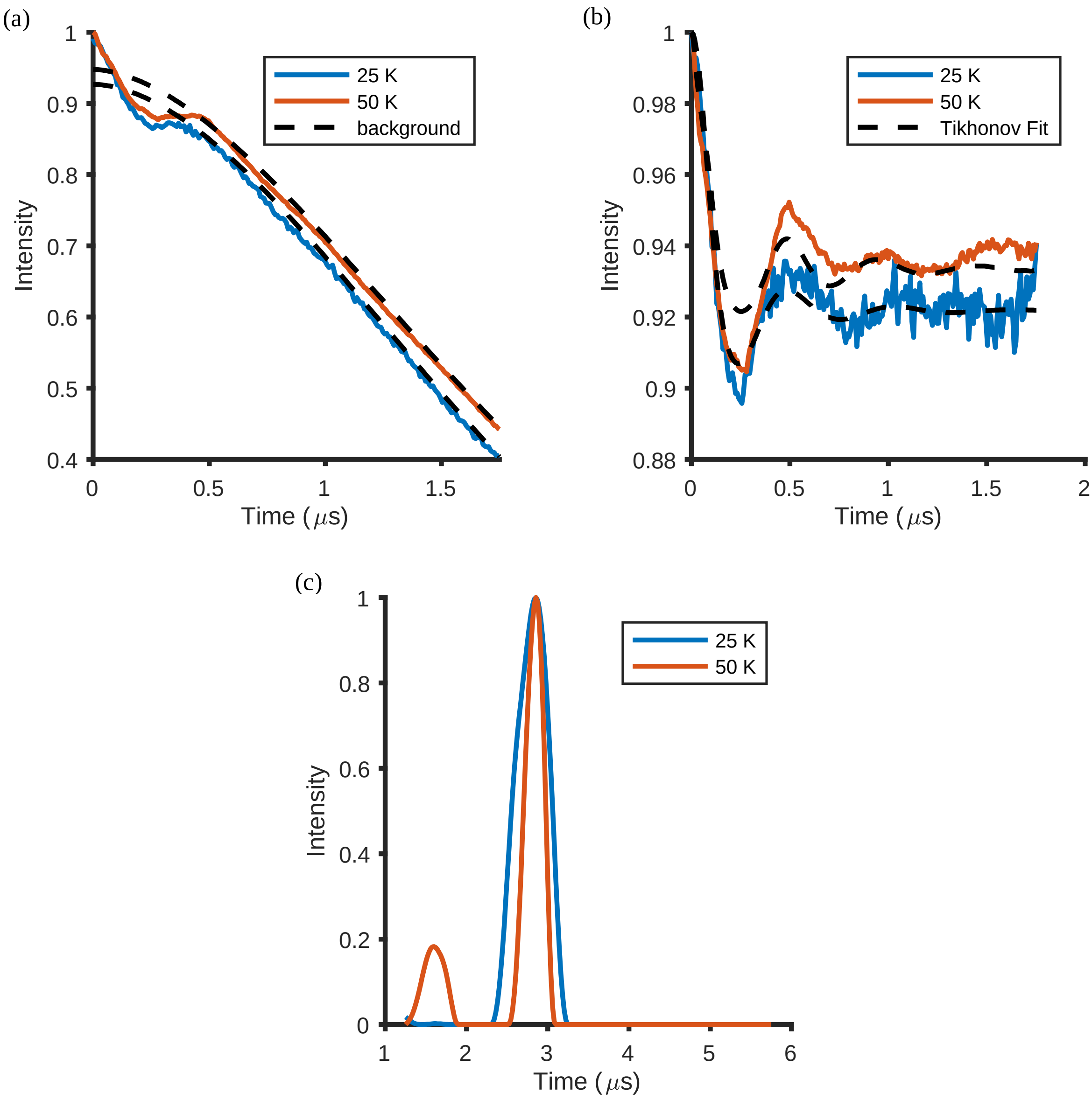


**Fig. S6** RIDME data measured at 25 K and 50 K cut to 1750 ns before background correction and Tikhonov regularisation fitting: (a) shows the raw RIDME data with accompanying stretched exponential background fitting, (b) is the background corrected RIDME traces cut to 1750 ns with the Tikhonov regularisation fit, and (c) is the accompanying extracted distance distributions

The 50 K data is shown to include an artefact, which is not present in the 25 K data, at around 1.5 nm. This artefact is likely the result of some distortion present at the beginning of the raw data which can be seen in Fig. S5a.

## S5. AGAO+Cu DEERS Measurements

Three- and four-pulse DEER experiments were carried out at 15 K on AGAO+Cu as defined in the main paper. In Fig. S7, two DeerStitch (DEERS) spectra are shown. [1] The overlap was 1025 ns, and modulation depth fitting was not used. The DEERS either has 80 ns of the four-pulse DEER used to replace the unmeasured (since it would suffer distortions) three-pulse DEER (“RepShort”), or uses the full length of the four-pulse measurement (“RepLong”). In the plots, it is observed that the results from DeerAnalysis2019 [2] for both iterations of the spectra overlap well.

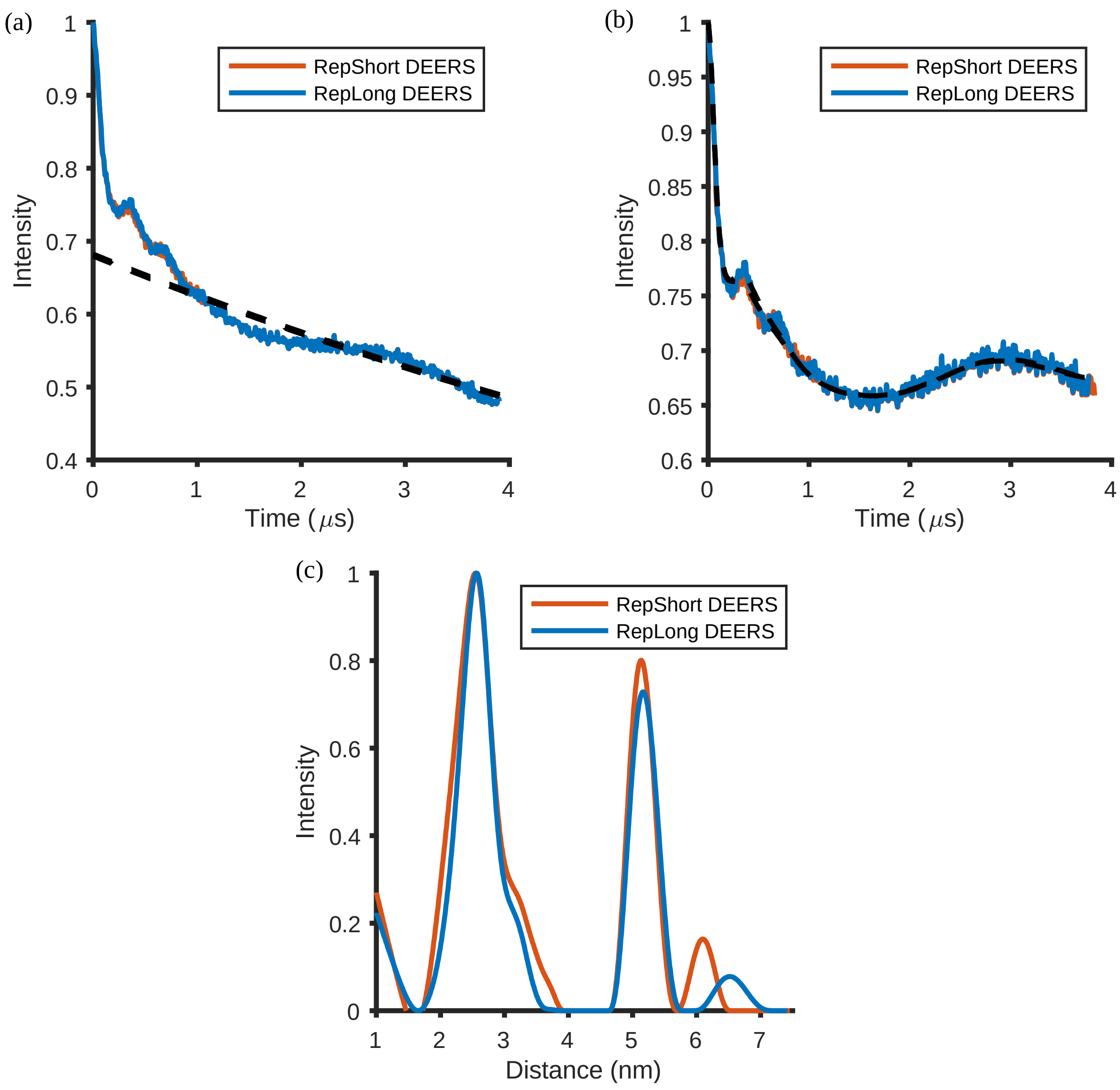


**Fig. S7** A comparison of the results from DeerAnalysis2019 for the RepShort and RepLong DEER-Stitch approaches, defined as indicated in the text, for AGAO+Cu: (a) shows the raw data for both the DEERS methods. The background of each spectrum is also presented as a dashed line on these plots; (b) provides the accompanying background corrected data. The background of both datasets were fit with a three-dimensional homogeneous exponential function. The RepShort data used a background start value of 588 ns, as determined by DeerAnalysis, and RepLong used 584 ns. In both cases, the zero time was 0 ns. Here, the Tikhonov regularisation fit is also presented as a dashed line on each spectrum; (c) provides the distance distributions extracted from the measurements after background correction and using Tikhonov regularisation methods. DeerAnalysis determined the Tikhonov regularisation parameter for both datasets using the L curve criterion. For both RepShort and RepLong, the parameter was 3980

## S6. AGAO+Cu RIDME Measurements

### S6.1 Effect of temperature on measurements

25 K, 30 K, and 40 K were trialled as potential measurement temperatures for the RIDME experiments. All three measurements consist of 1 scan and used time delays of $\tau_1$ = 200 ns and $\tau_2$ = 4280 ns, which were stepped 3 times in increments of 40 ns. The SRT was set to 15 ms, 8 ms, and 4 ms for 25 K, 30 K, and 40 K, respectively. The $T_1$ relaxation measurements of the Cu(II) spins were recorded using the

inversion recovery experiment at the three temperatures. In all cases, time delays of τ = 200 ns and T = 400 ns were used. For the 25 K measurement, T was stepped in increments of 400 ns, for 30 K and 40 K, this value was 300 ns. All three measurements used a SRT of 5 ms. Echo-decay measurements were also recorded at the three temperatures. The two-pulse sequence was used at a field position of 12109 G (nitroxide), and used π/2- and π-pulse lengths of 16 and 32 ns, respectively. The time delay was τ = 200 ns, stepped in increments of 10 ns. For the 25 K and 40 K measurements, a SRT of 75 ms was used, while the 30 K experiment used 40 ms. The inversion recovery curves of the Cu(II) spins, echo-decay curves of the nitroxide, and the raw RIDME data for each of these three temperatures, measured with $T_{mix}$ = 5 μs, are presented in Fig. S8.

The raw data in Fig. S8b shows the background of the RIDME signal to be largely unaffected by the change in temperature. The modulation depth improves from 25 to 30 K as expected from the inversion recovery curves (Fig. S8a), though this trend does not continue for the 40 K measurement. There is a large increase in noise associated with the 40 K measurement, presumably due to the phase memory limit of the nitroxide (Fig. S8b). We therefore concluded that a RIDME measurement temperature of 30 K is suitable for the AGAO+Cu RIDME measurements.

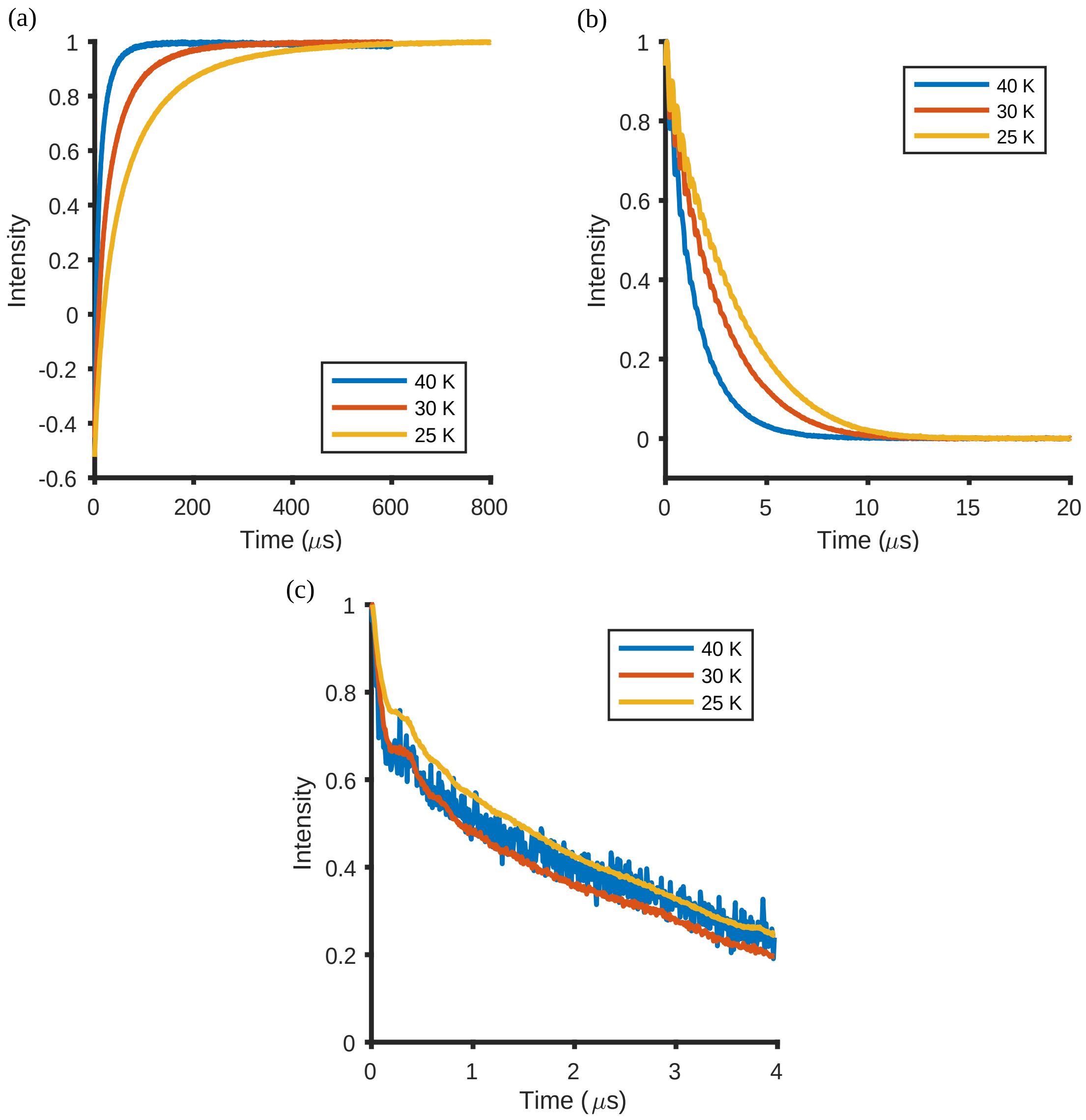


**Fig. S8** AGAO+Cu RIDME with $T_{mix}$ = 5 μs at 25, 30 and 40 K: (a) the inversion recovery curves of Cu(II), measured at a field position of 11814 G; (b) echo-decay curves of nitroxide, measured at a field position of 12109 G, and (c) the RIDME experiment data

## S6.2 Sample dilution

$T_1$ relaxation measurements were conducted for both the original (AGAO+Cu) and 10x diluted samples ($(AGAO+Cu)_{10xdil}$), the results are shown in Fig. S9. The three-pulse inversion recovery experiment was again used as before and was applied at the maximum field position of the nitroxide or Cu(II) peaks in the associated ED-FS (12109 G and 11814 G, respectively). AGAO+Cu experiments were conducted at 30 K. Pulse lengths of 16 ns and 32 ns were used for the π/2- and π-pulses, respectively, and time delays of $\tau$ = 200 ns and T = 400 ns, which was stepped in increments of 10 μs for nitroxide and 300 ns for Cu(II). SRT values of 40 ms and 5 ms were used for nitroxide and Cu(II), respectively. The $(AGAO+Cu)_{10xdil}$ experiments were identical, except that a SRT of 75 ms was used for the nitroxide measurement. Phase memory time measurements were conducted at a field position of 12109 G (the nitroxide) using the echo-decay experiment at 30 K. Pulse lengths of 16 ns and 32 ns were used for the π/2- and π-pulses, respectively, and the time delay was $\tau$ = 200 ns stepped in increments of 10 ns. For these experiments, a SRT of 40 ms and 75 ms were used for the AGAO+Cu and $(AGAO+Cu)_{10xdil}$ measurements, respectively.

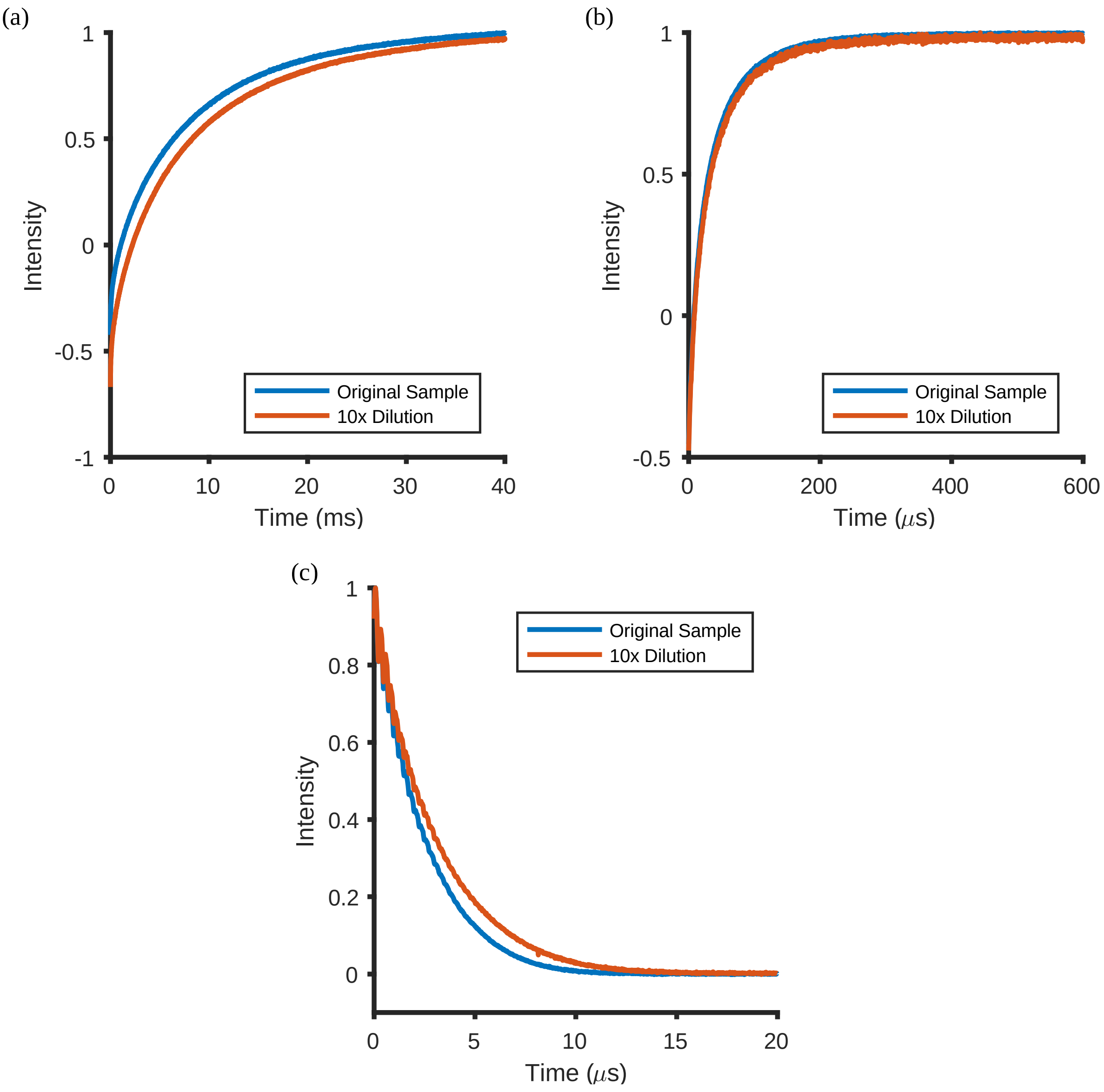


**Fig. S9** $T_1$ relaxation measurements from the AGAO+Cu sample at 30 K before and after protein concentration was reduced by 10 times: (a) nitroxide; (b) Cu(II). Phase memory relaxation measurements at 30 K for each sample are shown in (c)

Fig. S10 shows the raw time traces, their associated background corrected traces, and distance distribution results as measured from identical five-pulse RIDME experiments on both the AGAO+Cu and (AGAO+Cu)$_{10xdil}$. Both measurements were recorded at 30 K, used Q-band frequency, and had π- and π/2-pulses lengths of 32 ns and 16 ns, respectively. For the AGAO+Cu measurement, time delay values of $\tau_1$ = 200 ns and $\tau_2$ = 4280 ns were used. The (AGAO+Cu)$_{10xdil}$ measurements used $\tau_1$ = 200 ns and $\tau_2$ = 4280 ns. In both measurements, $\tau_1$ and $\tau_2$ were stepped 3 times in increments of 40 ns, the mixing time was 5 μs, the SRT was 8 ms, and the spectra were collected over 1 scan.

Both the AGAO+Cu and (AGAO+Cu)$_{10xdil}$ backgrounds were corrected by fitting with a stretched exponential function. The stretch parameters were determined by DeerAnalysis to be 3.33 and 2.31 dimensions for the AGAO+Cu and (AGAO+Cu)$_{10xdil}$ samples, respectively. The AGAO+Cu sample used a background start of 784 ns and a zero time of 137 ns, as determined by DeerAnalysis. The Tikhonov regularisation parameter for this sample was determined by DeerAnalysis to be 316 using the L curve criterion. The (AGAO+Cu)$_{10xdil}$ sample had a background start of 1096 ns, a zero time of 136, and a Tikhonov regularisation parameter of 631. No data was cut from either sample.

While there are some changes in the background function and the signal-to-noise through the dilution, the distance distribution results are quite stable.

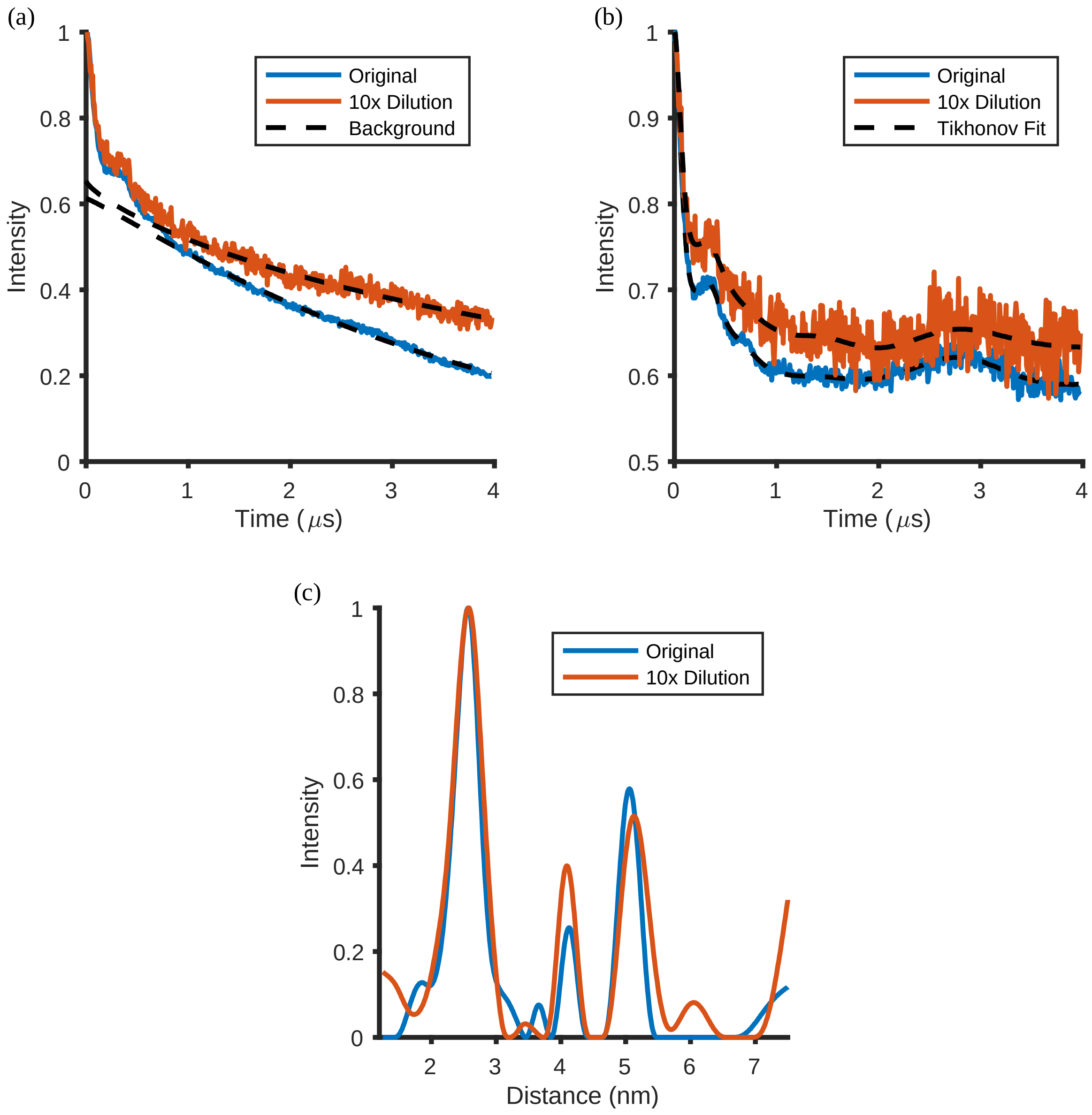


**Fig. S10** RIDME results from DeerAnalysis2019 for AGAO+Cu and $(AGAO+Cu)_{10xdil}$: (a) time traces with stretched exponential background function (dashed line); (b) background corrected; (c) distance distributions from Tikhonov regularisation

## S6.3 Effect of increasing Cu(II) concentration

To identify the effect of increased Cu(II) concentration on the modulation depth of the RIDME measurement, two new samples were produced from the $(AGAO+Cu)_{10xdil}$ sample; these were $(AGAO+Cu_2)_{10xdil}$ and $(AGAO+Cu_{12})_{10xdil}$, which refer to double Cu(II) and 12x Cu(II) concentration, as compared to $(AGAO+Cu)_{10xdil}$, respectively.

Inversion recovery measurements of Cu(II) (Fig. S11a) were recorded at 30 K, and at a field position of 11814 G. These experiments used the three-pulse sequence $\pi - T - \pi/2 - \tau - \pi - \tau -$ echo, with pulse lengths of 32 ns and 16 ns for the $\pi$- and $\pi/2$-pulses, respectively, and time delays $\tau$ = 200 ns and T = 400 ns, which was stepped in increments of 300 ns. A SRT of 5 ms was used. To measure the echo-

decay of the nitroxide, the two-pulse sequence $\pi/2 - \tau - \pi - \tau -$ echo was used at a field position of 12109 G, where the $\pi/2$- and $\pi$-pulse lengths were 16 and 32 ns, respectively, and the time delay was $\tau$ = 200 ns stepped in increments of 10 ns. For these experiments, a SRT of 75 ms was used.

Five-pulse RIDME experiments were conducted as described in the main paper text, with the exception that the $(AGAO{+}Cu)_{10xdil}$ and $(AGAO{+}Cu_2)_{10xdil}$ data were recorded with an SRT of 9180 ns, and 8160 ns was used for $(AGAO{+}Cu_{12})_{10xdil}$. In all three experiments, the data was collected over 1 scan. The raw RIDME data for the three measurements are presented and compared in Fig. S11c.

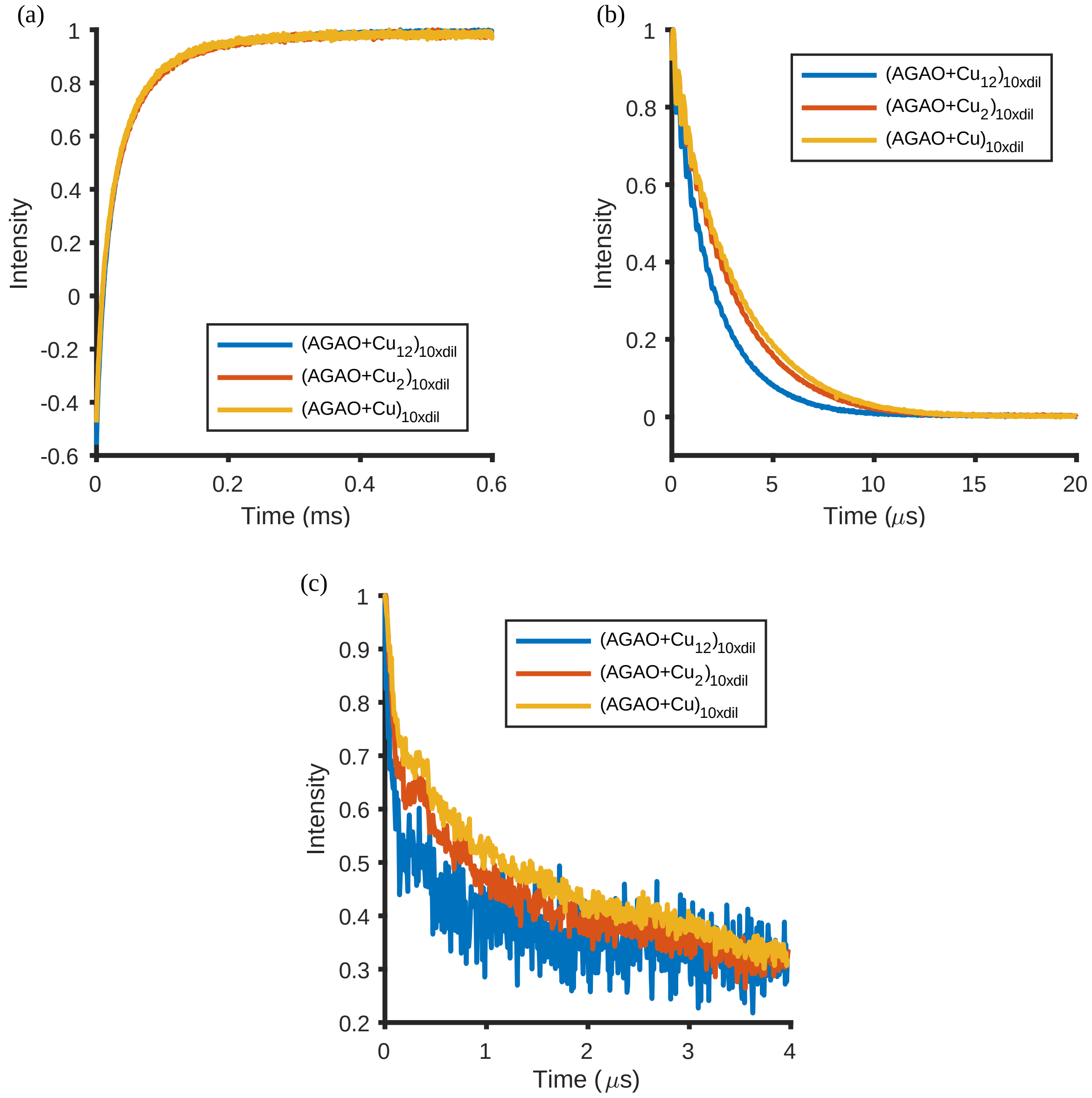


**Fig. S11** AGAO+Cu relaxation measurements and RIDME with $T_{mix}$ = 5 μs with increasing Cu(II) concentrations: (a) inversion recovery curves of Cu(II), measured at a field position of 11814 G; (b) echo-decay of nitroxide, measured at a field position of 12109 G; (c) the RIDME experiment data

Here we observe that the modulation on the echo increases with increasing copper ratio, but by a subtle amount. The signal-to-noise deteriorates as Cu(II) is added, and we do not analyse this data further.

## S7. Alternative RIDME fitting Methods

### S7.1 Stretched exponential vs polynomial background fitting

All of the RIDME data presented in the main paper text has been fitted with a stretched exponential background function. Here, we present it again alongside the data background fit with a low-order polynomial function. It has been shown that low-order polynomial functions can be used to fit the RIDME background. [3-5] For experiments run at temperatures of 30 K and under, and for $\tau_2$ values less than 1.5 μs, it may be preferable to use the low-order polynomial background fit. The comparison of fitted background function type for the distance distributions shown in the main paper are given below.

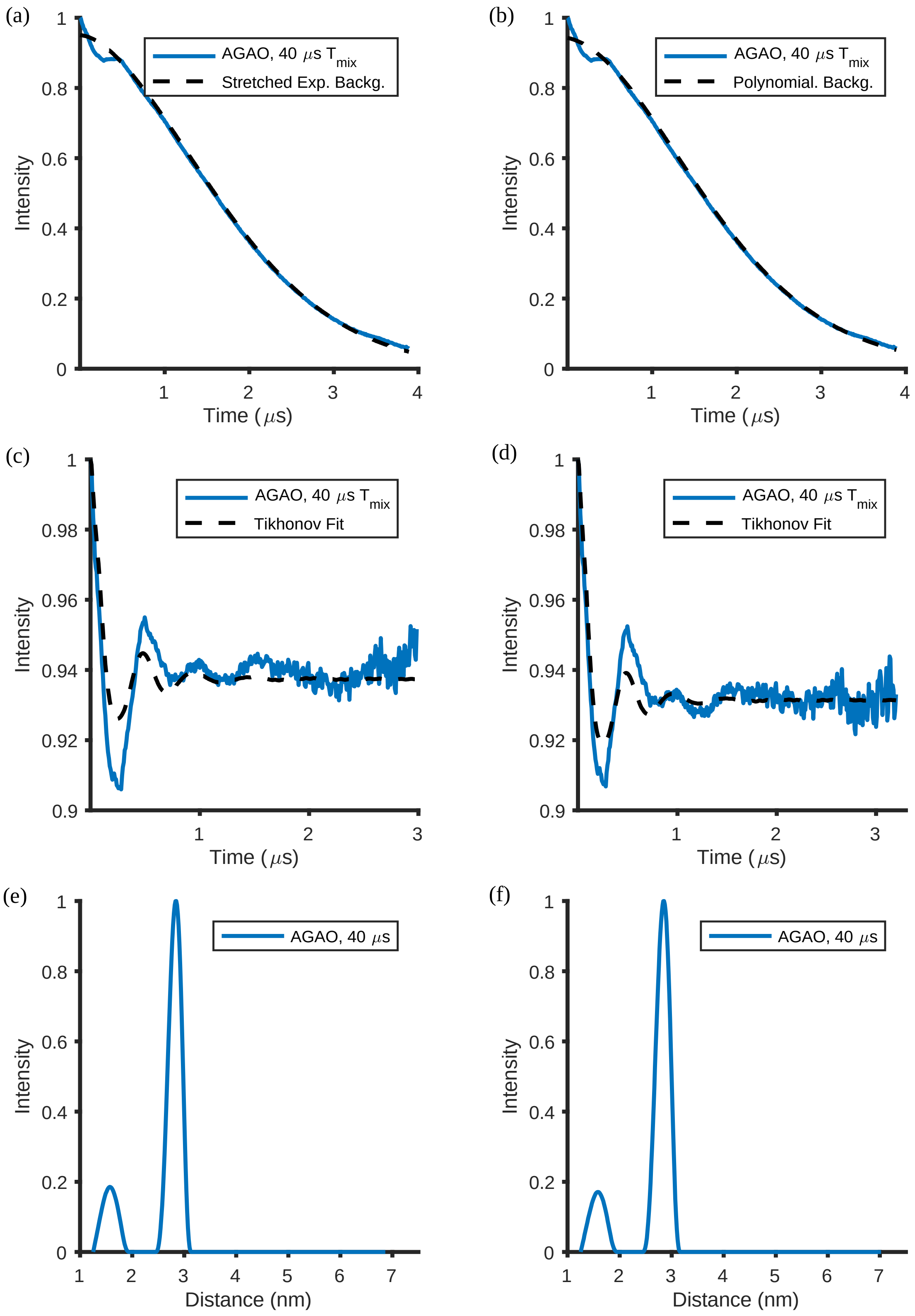


**Fig. S12** AGAO NO-NO RIDME at 50 K. Comparison of stretched exponential, with a DeerAnalysis determined stretch parameter of 5.13 dimensions, and third-order polynomial background fit. The stretched exponential fits (left) were carried out as described in the main text. The polynomial fit (right) used a DeerAnalysis-determined background start value of 656 ns, and a zero time of 341 ns. The Tikhonov regularisation parameter, determined by DeerAnalysis according to the L curve criterion, was 126

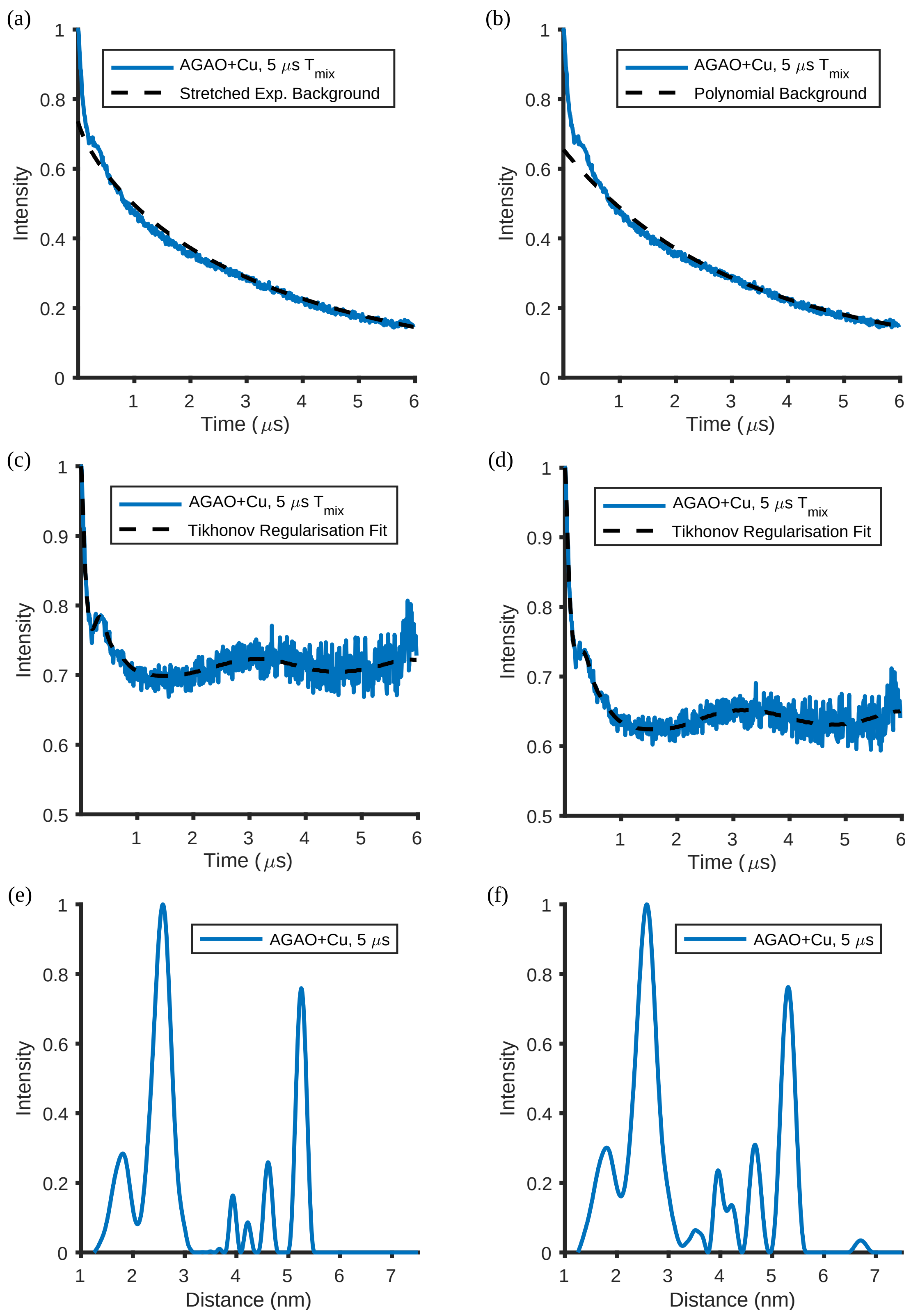


**Fig. S13** AGAO+Cu at 30 K with $\tau_2$ = 6280 ns and $T_{mix}$ 5 μs. Comparison of stretched exponential, with a DeerAnalysis determined stretch parameter of 2.37 dimensions, and second-order polynomial background fit. The stretched exponential fits (left) were carried out as described in the main text. The polynomial fit (right) used a DeerAnalysis-determined background start value of 1912 ns, and a zero time of 135 ns. The Tikhonov regularisation parameter, determined by DeerAnalysis according to the L curve criterion, was 501

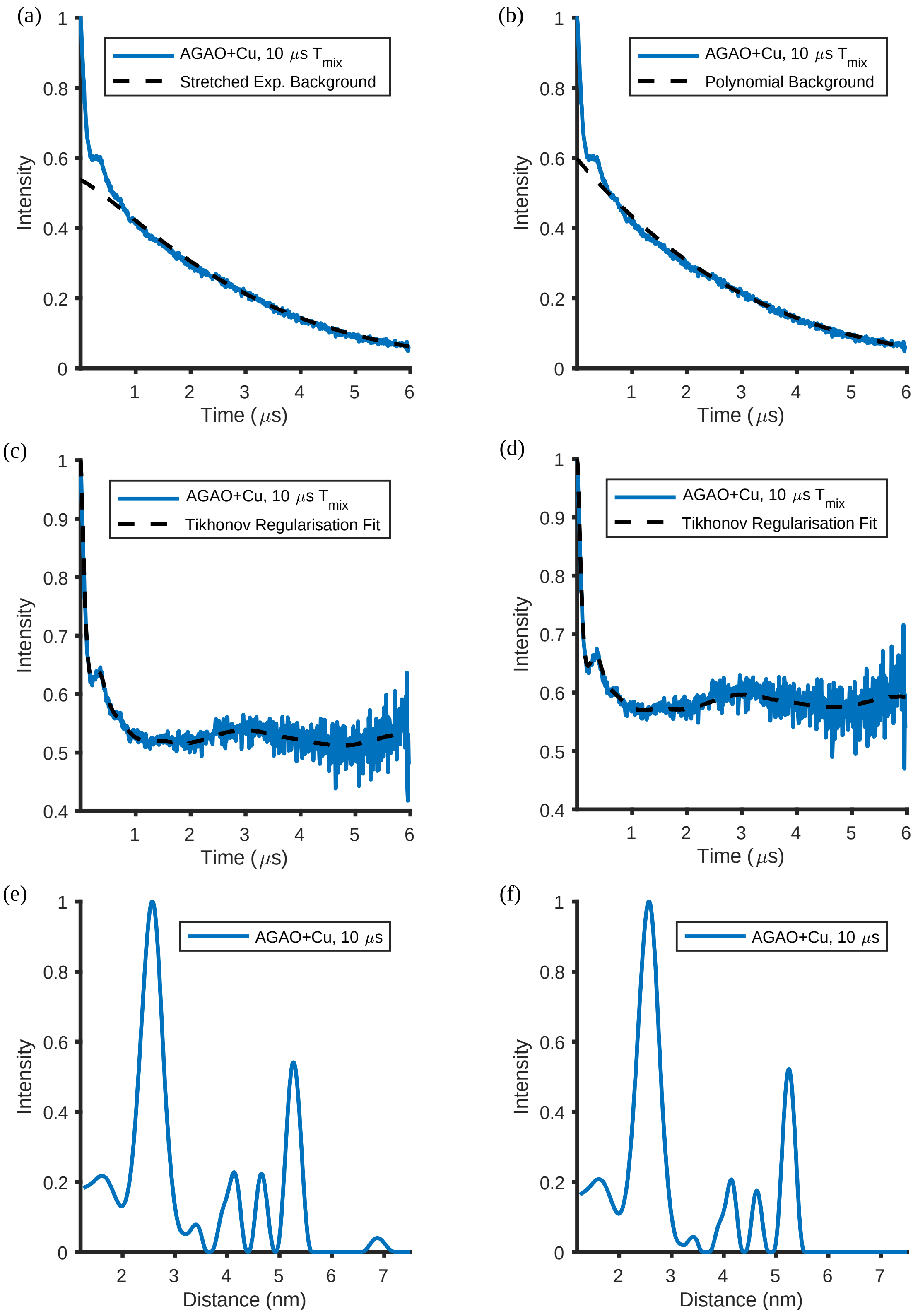


**Fig. S14** AGAO+Cu at 30 K with $\tau_2$ = 6280 ns and $T_{mix}$ 10 μs. Comparison of stretched exponential, with a DeerAnalysis determined stretch parameter of 6.31 dimensions, and second-order polynomial background fit. The stretched exponential (left) fits were carried out as described in the main text. The polynomial fit (right) used a DeerAnalysis-determined background start value of 1672 ns, and a zero time of 138 ns. The Tikhonov regularisation parameter, determined by DeerAnalysis according to the L curve criterion, was 631

## S7.2 Tikhonov regularisation vs Gaussian distribution analysis

In the main paper, all of the background corrected RIDME traces were fitted using Tikhonov regularisation. Here, we present the nitroxide-Cu RIDME results alongside those obtained through fitting with two and three Gaussians. [6, 7] To the best of our knowledge, DeerAnalysis Gaussian fitting models have not been used to analyse RIDME background corrected traces in published work, but Gaussian fitting of RIDME traces has been carried out using an alternative fitting package. [8] Here, we apply the DeerAnalysis Two_Gaussian and Three_Gaussian pre-installed models to our RIDME data. Figs. S15a and S15b show the traces of the nitroxide-Cu RIDME data measured at 30 K with a 5μs $T_{mix}$. Background correction was performed using stretched exponential and second-order polynomial models as described in section S7.1. A black dotted line shows the fit achieved using Tikhonov regularisation, while the red dotted line is that achieved by the fitting of two Gaussians. Figs. S15c and S15d show the accompanying distance distributions obtained via each method.

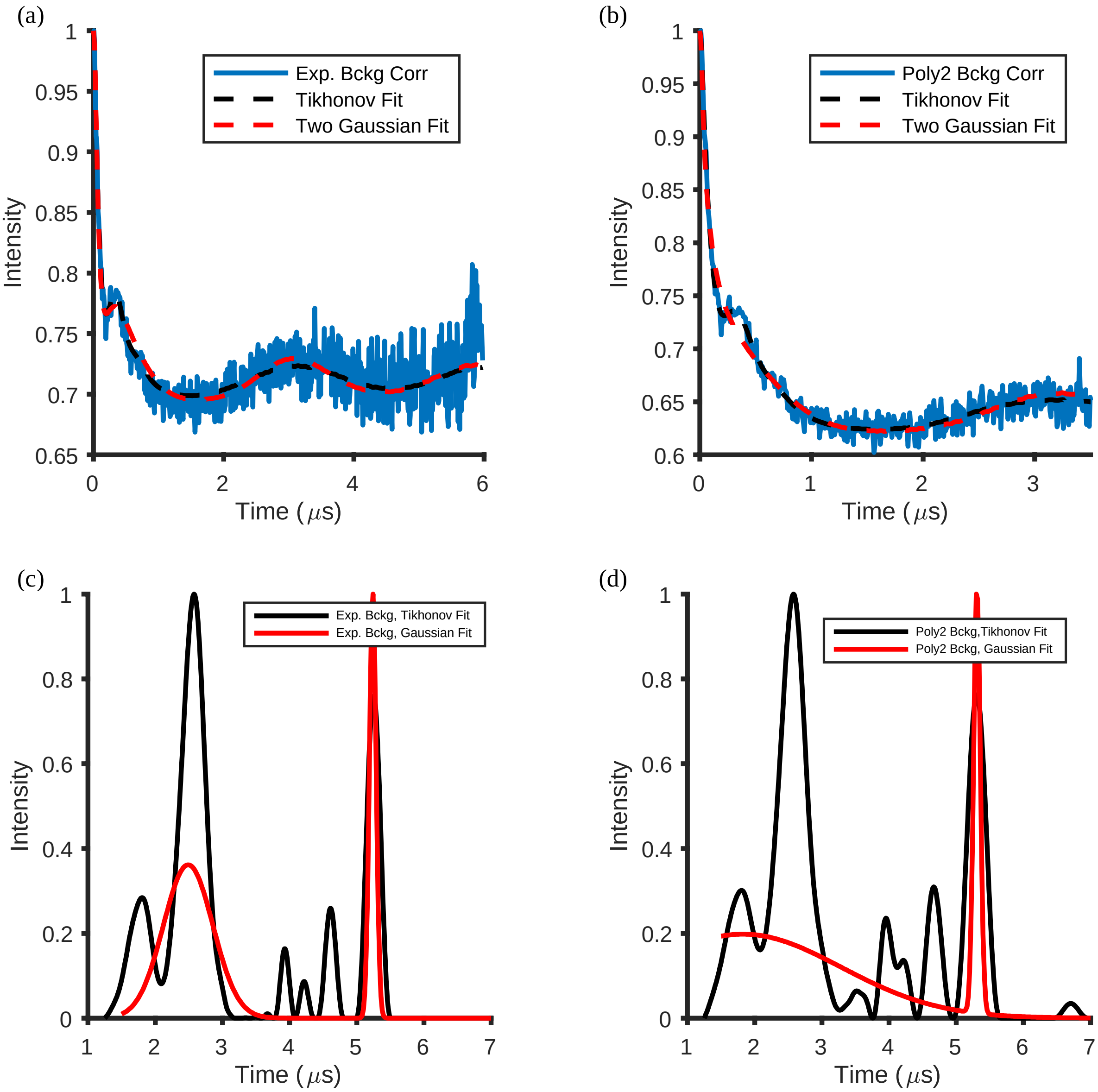


**Fig. S15** AGAO+Cu RIDME at 30 K with $\tau_2$ = 6280 ns and $T_{mix}$ 5 μs. Comparison of Tikhonov regularisation and Gaussian fitting methods. The background correction was carried out as described in section S7.1, the Tikhonov regularisation parameters were as presented in the main paper, and the Two_Gaussians model in DeerAnalysis was used to fit two Gaussian functions to the background corrected trace. The parameters of this fit were unconstrained: (a) and (c) show the RIDME time trace background corrected

using a stretched exponential function, with the Tikhonov and two Gaussian fits presented as dotted lines, and the accompanying extracted distance distributions, respectively; (b) and (d) show the RIDME time trace background corrected using a second-order polynomial function, again with the Tikhonov and two Gaussian fits presented as dotted lines, and the accompanying extracted distance distributions, respectively

For the trace background corrected by a stretched exponential (Fig. S15a), the two Gaussian fit provides a distance distribution with two peaks (Fig. S15c). The peak at a shorter distance has a mean distance of 2.49 nm with a standard deviation of 0.37 nm. The longer distance has a mean of 5.24 nm with a standard deviation of 0.05 nm. This is in good agreement with the distance values derived from the Tikhonov regularisation presented in the main paper.

The second-order polynomial background corrected RIDME trace fitted with two Gaussian functions (Figs S15b and S15d) provides less conclusive results. The shorter distance is represented by a broad peak which exceeds the bounds of the distribution, while the longer distance peak has a mean distance of 5.31 nm with a standard deviation of 0.05 nm.

Fig. S16 shows the background corrected RIDME traces and accompanying fit carried out using three Gaussian functions through DeerAnalysis's Three_Gaussians model, as well as the associated extracted distance distributions. The overall form of the distance distributions align better, regardless of background model, than for the two Gaussian fit (Fig. S15). Both resemble the Tikhonov-derived results well.

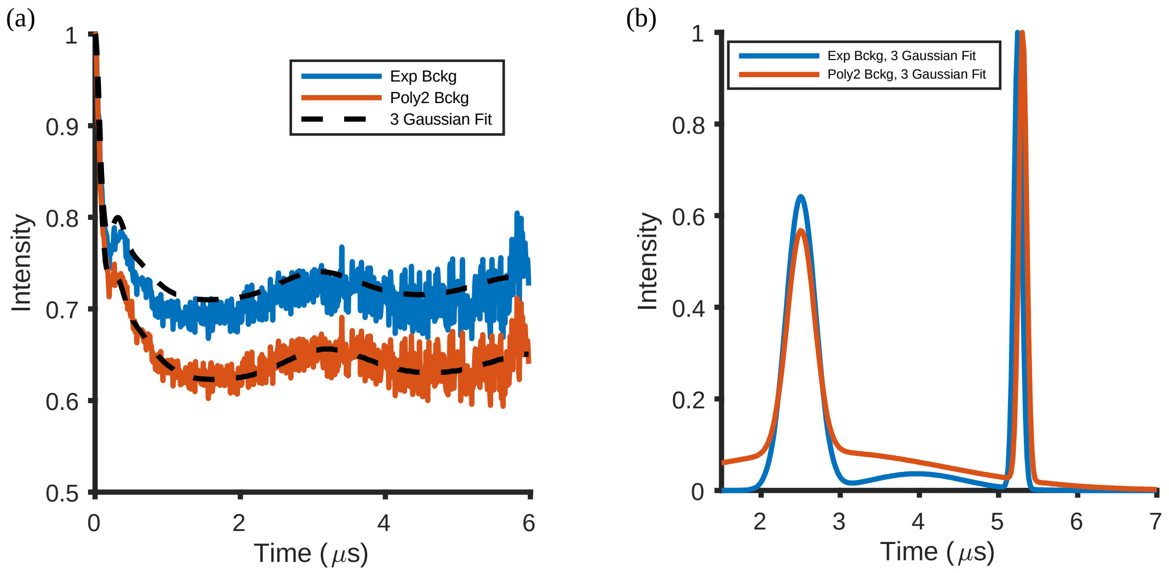


**Fig. S16** AGAO+Cu RIDME at 30 K with $\tau_2$ = 6280 ns and $T_{mix}$ 5 μs. Comparison of Tikhonov regularisation and Gaussian fitting methods. The background correction was carried out as described in section S7.1, the Tikhonov regularisation parameters were as presented in the main paper, and the Three_Gaussians model in DeerAnalysis was used to fit three Gaussian functions to the background corrected trace. The parameters of this fit were unconstrained: (a) shows RIDME traces background corrected using a stretched exponential and a second-order polynomial function. The three Gaussian fit is shown as a dotted line; (b) shows the accompanying extracted distance distributions from each of the traces in (a)

The RIDME trace background corrected using a stretched exponential (Fig. S16a) provides a short distance distribution peak (Fig. S16b) with a mean distance of 2.57 nm with a standard deviation of 0.11 nm. The longer distance has a mean of 5.24 nm with a standard deviation of 0.05 nm. There is a small contribution at middling distances. The second-order polynomial corrected trace fitted with three Gaussian functions (Fig. S16a) shows the most probable separation (Fig. S16b) of the short distance peak to be 2.42 nm with a standard deviation of 0.47 nm. There is an underlying broad distribution too.

The longer distance peak between 5.15 nm and 5.50 nm has a mean of 5.32 nm with a standard deviation of 0.06 nm.

## S8. Background Validation Data

Here we present the background validation data for each of the distance distributions presented in the main paper. Validation was carried out following Tikhonov regularisation in DeerAnalysis2019 [2]. The validation parameters were set such that fits were conducted over a range of background starting values. The background starting point minimum was around 5% of the total length of the time trace and the maximum was approximately 80 % for DEER data, and 50% for RIDME data, due to limitations produced by the RIDME spectra. A total of 16 trials between these two values were conducted to achieve the unpruned validation results shown in Figs. S17 and S18. In these figures, the distance distribution obtained via the fitting methods previously described are shown as a solid blue line, and the associated DeerAnalysis-determined confidence intervals are shown in grey.

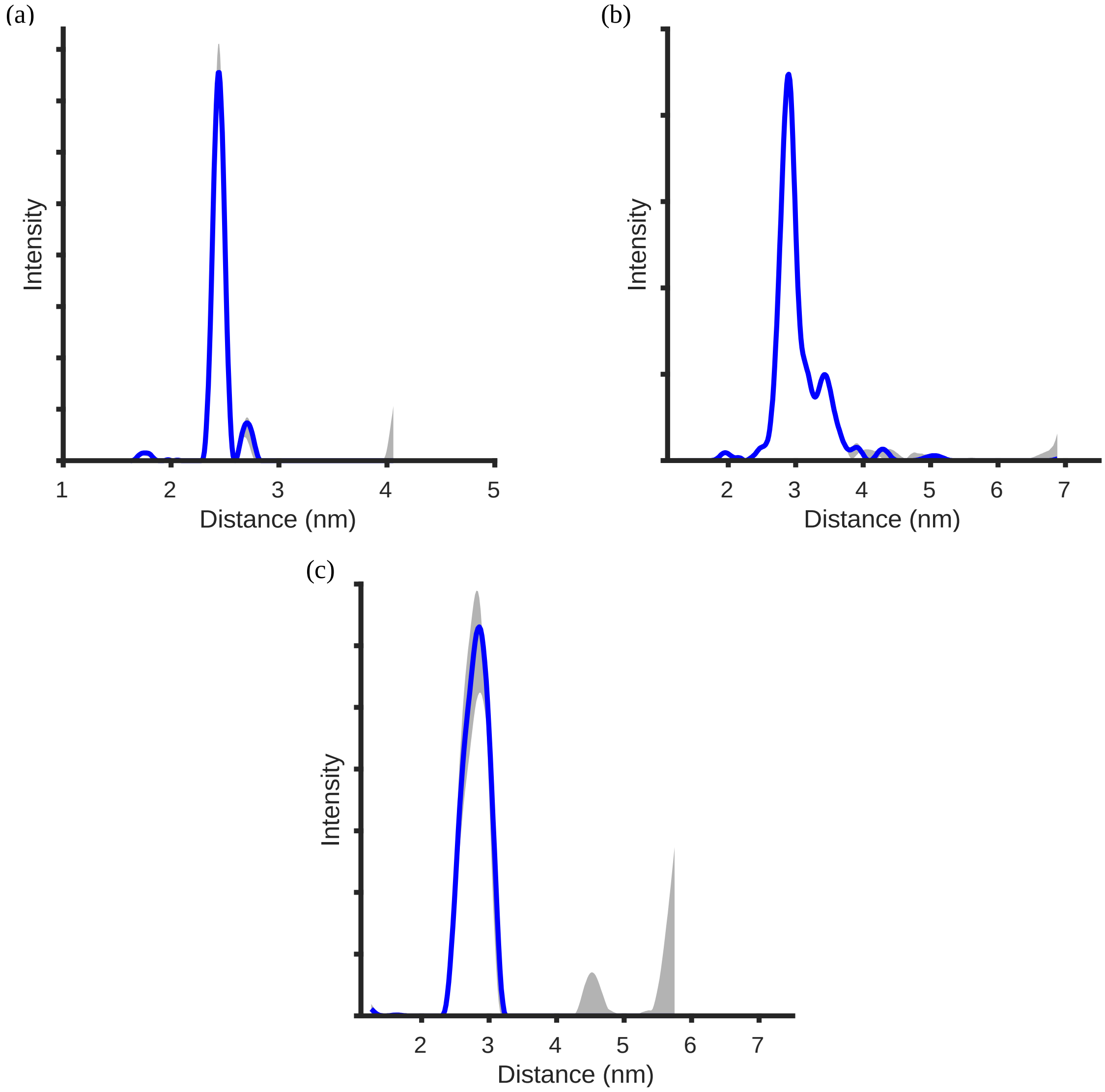


**Fig. S17** Validation data for each of the AGAO DEER and RIDME measurement results presented in the main paper; (a) AGAO X-band DEER; (b) AGAO Q-band DEER; (c) AGAO 25 K, $T_{mix}$ = 40 μs RIDME

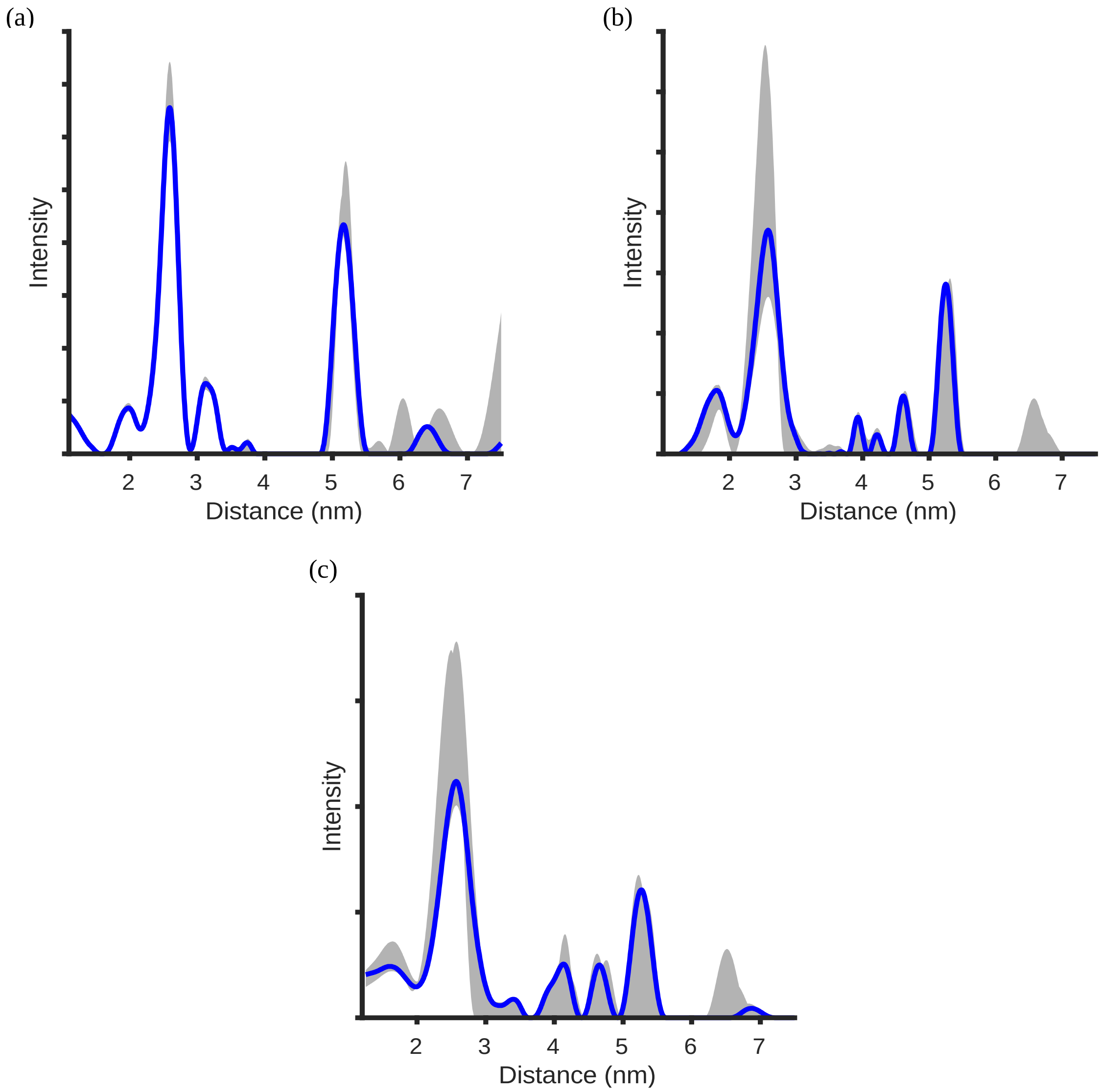


**Fig. S18** Validation data for each of the AGAO+Cu DEER and RIDME measurement results presented in the main paper: (a) AGAO+Cu RepLong DEERS; (b) AGAO+Cu 30 K, $T_{mix}$ = 5 µs RIDME measured over 25 scans; (c) AGAO 30 K, $T_{mix}$ = 10 µs RIDME measured over 25 scans

From the validation of the AGAO+Cu RIDME background, it is suggested that the peak in the distribution with a most probable distance of 5.31 nm in Fig. S18b, and 5.26 nm in Fig. 18c, are not robust to the choice of background starting value. However, we can be confident that it is a real distance as Fig. S18a shows the DEERS measurement of the same sample. DEERS has a far less severe background than RIDME which leads to a more stable background correction.

## S9. MD Simulation AGAO Modelling

Fig. S19 shows the nitroxide-nitroxide distance separations calculated over 100 ns of MD simulation at a temperature of 273 K. The mean nitroxide-nitroxide separation during the simulation was 3.10 nm, with a standard deviation of 0.37 nm.

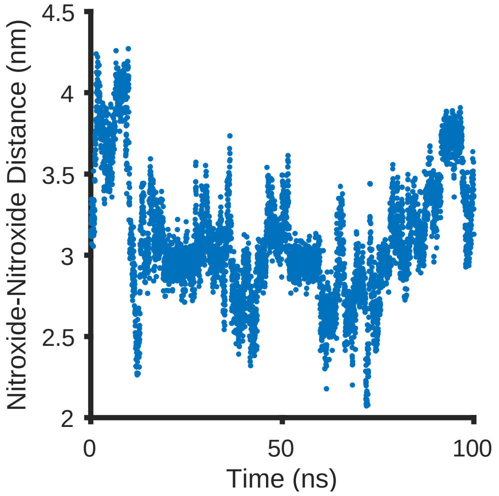


**Fig. S19** Nitroxide-nitroxide distances calculated over 100 ns of MD simulation. The mean separation was 3.10 nm with a standard deviation of 0.37 nm.

## References


1. J.E. Lovett, B.W. Lovett, and J. Harmer, J. Magn. Reson. **223,** (2012): pp. 98-106.
2. G. Jeschke, V. Chechik, P. Ionita, A. Godt, H. Zimmermann, J. Banham, C.R. Timmel, D. Hilger, and H. Jung, Appl. Magn. Reson. **30,** 3 (2006): pp. 473-498.
3. A. Giannoulis, C.L. Motion, M. Oranges, M. Bühl, G.M. Smith, and B.E. Bode, Phys. Chem. Chem. Phys. **20,** 4 (2018): pp. 2151-2154.
4. D. Abdullin, F. Duthie, A. Meyer, E.S. Müller, G. Hagelueken, and O. Schiemann, J. Phys. Chem. B. **119,** 43 (2015): pp. 13534-13542.
5. I. Ritsch, H. Hintz, G. Jeschke, A. Godt, and M. Yulikov, Phys. Chem. Chem. Phys. **21,** 19 (2019): pp. 9810-9830.
6. Y. Song, T.J. Meade, A.V. Astashkin, E.L. Klein, J.H. Enemark, and A. Raitsimring, J. Magn. Reson. **210,** 1 (2011): pp. 59-68.
7. M. Teucher, M. Qi, N. Cati, H. Hintz, A. Godt, and E. Bordignon, *Orthogonally spin-labeled rulers help to identify crosstalk signals and improve DEER signal fidelity*. 2020.
8. D. Abdullin, P. Brehm, N. Fleck, S. Spicher, S. Grimme, and O. Schiemann, Chem. Eur. J. **25,** 63 (2019): pp. 14388-14398.
